# Production efficiency and GHG emissions reduction potential evaluation in the crop production system based on emergy synthesis and nonseparable undesirable output DEA: A case study in Zhejiang Province, China

**DOI:** 10.1371/journal.pone.0206680

**Published:** 2018-11-01

**Authors:** Gang Dong, Zhengzao Wang, Xianqiang Mao

**Affiliations:** 1 School of Environment, Beijing Normal University, Beijing, China; 2 Economics Research Institute, Anhui University of Finance and Economics, Bengbu, China; Wageningen University, NETHERLANDS

## Abstract

Maintaining crop outputs to feed its large population with limited resources while simultaneously mitigating carbon emissions are great challenges for China. Improving the efficiency of resource use in crop production is important in reducing carbon emissions. This paper constructs a methodological framework combining emergy-based indicator accounting and a nonseparable undesirable output slack-based measurement (SBM) data envelopment analysis (DEA) model. This framework is used to explore the efficiency of inputs and outputs and the greenhouse gas (GHG) emissions reduction potential for crop production systems, using Zhejiang province, China, as a case study. It is found that an emergy synthesis and a nonseparable undesirable output SBM-DEA framework is compatible with the case study. Crop production in Zhejiang province has relied heavily on an increase in agrochemical inputs to maintain agricultural output. Energy and chemical fertilizer use are determined as the province’s major carbon emissions sources. Although carbon emissions per unit of monetary output has decreased sharply, the carbon emissions per unit emergy output has increased, demonstrating a high carbon intensity reality. The DEA highlighted the differences in crop production efficiency, resource factor redundancy and carbon mitigation potential in the different prefectures of the province. To conclude this research, policies to support low carbon agriculture development, including subsidizing low carbon agriculture technology development and expansion and the cancellation of subsidies to high carbon production factors, such as chemical fertilizer production and sales, are discussed to conclude the research.

## 1. Introduction

The agricultural sector accounts for almost 14% of global net carbon emissions [1]. In China, rapid industrialization, urbanization and land-use changes have led to a reduction in cultivated land across China over the past few decades [2]. To increase crop productivity and sustain its large population, the conventional approach in China is to intensively apply high carbon inputs, such as fertilizer, agricultural machinery, fuels and other industrial products. However, this heavy reliance on high carbon inputs has caused large direct and indirect greenhouse gas (GHG) emissions in the agricultural sector [3]. In China, agricultural production accounted for 11% of total carbon emissions (or approximately 820 MtCO_2-eq_ per year), over 70% of total nitrous oxide (N_2_O) emissions and approximately 50% of methane (CH_4_) emissions in 2005 [4].

Potential carbon reduction largely depends on improving efficiency. Commonly, when considering agricultural production efficiency, economic dimensions have been a priority. However, such production efficiency evaluations based on economic indicators might overestimate efficiency and cause a “low carbon illusion”. Thus, production efficiency evaluations based on energy or emergy accounting and classifying direct and embodied carbon or GHG emissions as nonseparable undesirable outputs have become essential elements in the evaluation of the sustainability of agriculture practices; these evaluations may generate important implications for decision makers.

Based on emergy analysis and a nonseparable undesirable output slack-based-measurement (SBM) DEA model [5], this paper constructs an analytical framework to evaluate the efficiency performance of crop production systems, in order to disclose the high carbon reality and the carbon reduction potential in the crop production system and therefore provide policy implications for low carbon agriculture development.

Zhejiang province, located on China’s southeast coastline, covers a relatively small total area of 101,800 square kilometers, making it one of the smallest provinces in China. However, the province has a dense population. The area consists of mountainous and hilly areas (70.4%), plains and basins (23.2%), and lakes, rivers and reservoirs (6.4%). As an economically developed area, the agricultural sector of the province intends to develop a high-efficient crop production model. However, to determine whether said model can produce verifiably low carbon emissions, a diagnostic study is necessary.

Section 2 of this paper presents a carbon efficiency evaluation method based on emergy and the nonseparable undesirable output SBM-DEA. Section 3 provides the carbon efficiency evaluation of crop production systems at the provincial and prefecture-levels in Zhejiang province. The low carbon illusion and the high carbon reality, as well as the carbon reduction potential for the province and different prefectures, are analyzed. Finally, Section 4 concludes this paper and provides policy implications for sustainable low carbon agriculture development in Zhejiang province and across the nation.

## 2. Methods and data

The literature on efficiency measurement has proposed at least three commonly used methods. One is an indicator approach based on physical and monetary resource inputs and outputs and environmental impacts [5]. However, the consideration of traditional resource inputs in efficiency accounting was incomplete, especially for free natural resources [6]. Odum [7] argued that embodied energy, or emergy, whose formation follows the laws of thermodynamics, should be considered for free natural resources. Monetary accounting systems do not comply with these laws, nor can they link economic systems with natural ecosystems; thus, these systems experience a difficulty in estimating natural and economic value [8,9]. In contrast, an emergy analysis, rooted in thermodynamics and system ecology theory, can evaluate all resource input and product output flows and the resource use efficiency in a common biophysical unit [7,10]. In this paper, the amount of carbon equivalents (CO_2-eq_) per unit of emergy or monetary output is employed as the efficiency indicator [11,12].

The second method is a nonparametric data envelopment analysis (DEA) introduced by Charnes et al. [13]. This method can be used to comprehensively evaluate the total-factor efficiency and considers all inputs and desirable and undesirable outputs [14–18]. The DEA approach of the mathematical programming does not require the construction of specific function forms, which avoids making arbitrary assumptions of the distributional form [19]. DEA usually assumes that producing more outputs relative to less resource inputs and less undesirable outputs (environmental impacts) is a criterion of efficiency [20,21]. Normally, if no undesirable output (or environmental impacts) exists, then a slack-based measurement (SBM)-DEA, which deals with input reduction and output expansion at the same time and is more in line with reality, should be used [22]. If an undesirable output exists, then a normal undesirable output SBM-DEA should be applied [5]. However, multiple studies have observed that some undesirable outputs cannot be separated from inputs and the corresponding desirable outputs, in which case, a nonseparable undesirable output (SBM)-DEA model should be employed [20,23,24].

The third commonly used method is the parametric approach stochastic frontier analysis (SFA), which was independently proposed by Aigner, Lovell and Schmidt in 1977 [25,26,27]. The SFA model, which has the virtue of being stochastic and takes random errors into account, can distinguish the effects of statistical noise from those of productive inefficiency items. In most cases, both methods (DEA and SFA) achieve highly correlated results [28]. However, the parametric method can compound the problem of misspecification of functional forms. In addition, a flexible form is susceptible to multicollinearity, and theoretical restrictions may be violated [29]. For the present research, observational data were limited to 11 prefectures over 5 years. A parametric method application test showed strong multicollinearity within the four explanation variables (See Table A in S1 File). Thus, the SFA method was not applied in this paper.

### 2.1 Inputs use efficiency indicators based on emergy and carbon accounting

Emergy, developed from thermodynamics and systems ecology in the 1980s, is defined as the energy embodied in any form of goods or services. It is sometimes thought of as energy memory to evaluate different energy, material, services, etc. in terms of a single energy type of solar joules (sej) [7,30]. Emergy is proposed as an indicator of aggregate resource use for life cycle assessments (LCA) [31]. Compared to the traditional LCA method that measures downstream environmental burdens, emergy accounting boundaries are extended to incorporate all inputs [32], including sunlight, rain, wind, and heat, that contribute to agriculture production. The emergy accounting method is used in agricultural production systems with two kinds of analysis: bottom-up and top-down. Bottom-up emergy analysis usually quantifies the resources required at the farm level for different agricultural products [33]. Top-down emergy analysis approaches the farming system holistically to analyze the contributions of various natural resources to agricultural production [34]. An advantage of the latter is that it makes use of socioeconomic statistical datasets and can conduct time series analyses. This paper uses the top-down approach.

In this study, there are four conceptual categories of production inputs accounted for in emergy values, namely, free renewable natural resources (FRR), free nonrenewable natural resources (FNR), purchased renewable resources (PRR) and purchased nonrenewable resources (PNR), as described in **Table 1**. Crop output (Y^em^) is also accounted for in the study in terms of emergy. The emergy accounting data sources used for inputs and outputs are listed in the right column of **Table 1**.

**Table 1:** Data sets of input and output based on emergy and carbon accounting.

Category	Resource flows	Data sources
Free renewable natural resources (FRR)	Sunlight, rain, wind, earth cycle	All emergy calculations in this paper are based on the 15.83E+24 sej/y emergy baseline [20]. Other transformities are referenced from Brown and Ulgiati [35], Odum [20], Bastianoni et al. [36]. The inputs and outputs of agricultural production of Zhejiang province were calculated based on the analysis of crop commodities and the inputs of crop production systems for the period 1978–2014 from the following documents: Zhejiang Statistical Yearbooks [37] Comprehensive Agricultural Statistical Data and Material on 60 Years of New China [38] National Compilation of Data of Agricultural Product Cost and Revenue (1953–1997) [39] China Agricultural Products Cost-Benefit Yearbook [40] GHG emission factors were drawn from the IPCC [41] and all emissions of GHGs were measured with the carbon dioxide equivalent.
Free nonrenewable natural resources (FNR)	Net loss of topsoil	
Purchased renewable resources input (PRR)	Irrigation water, labor and services	
Purchased nonrenewable resources input (PNR)	Mechanical equipment, chemical fertilizers, pesticides, plastic mulch, energy resources	
Desirable crop outputs (Y^em^)	Rice, wheat, corn, soybean, tubers, fruits, Vegetables, Crop residues, and various economic crops	
Carbon emissions (or undesirable outputs) (CO_2-eq_)	GHG emissions of CO_2_, CH_4_, N_2_O	

Note: Detailed data sets for this table please see Table B in S1 File.

In this table, carbon emissions (or undesirable outputs) from the crop production include CO_2_, CH_4_, and N_2_O. Although carbon emissions from agricultural use energy production and consumption (diesel, electricity power, and energy embodied in agricultural chemicals) are accounted for as emissions from energy sectors in IPCC guidelines [41], in this study, these carbon emissions are categorized as emissions from the agricultural sector to reflect agricultural production’s complete carbon footprint.

The calculation of direct and indirect CO_2_ emissions is based on the research of Dong et al. [42] and Bennetzen et al. [43]. The energy input is partitioned into three categories: primary energy consumed directly during crop production, such as diesel consumed by machinery; secondary energy embodied in thermal power electricity; and tertiary energy embodied in fertilizers, pesticides and films.

All GHG emissions are converted to their CO_2_ equivalent (CO_2-eq_) using their 100-year global warming potentials (GWPs): 1 for CO_2_, 25 for CH_4_, and 298 for N_2_O [3].

In this study, local data at the provincial and prefecture levels in Zhejiang province were collected from the local statistical yearbooks (1995–2016) of Hangzhou, Ningbo, Wenzhou, Jiaxing, Huzhou, Shaoxing, Jinhua, Quzhou, Zhoushan, Taizhou and Lishui [44–54]. The emergy accounting-based indicators to characterize crop production efficiency, including total emergy input (U), emergy input density (EID), ecological environmental loading radio (ELR), purchased input ratio (PIR), self-sufficiency ratio (SSR) and emergy yield ratio (EYR), were constructed according to Odum [7].

The carbon emissions accounting and the monetary and emergy-based outputs accounting are combined to reflect multiple aspects of efficiency, including carbon-emergy output intensity (CemI) and carbon-monetary output intensity (CmI). The formulae and function description of these indicators are introduced and listed in **Table 2**.

**Table 2:** Emergy and carbon emissions-based production efficiency indicators.

Indicators	Formula	Description
Total emergy input (U)	U = (FRR+FNR+PRR+PNR)	Total resource emergy used
Emergy input density (EID)	EID = U/sown area	The indicator reflects the intensity of the total resources emergy flow input per unit sown area
Ecological environmental loading radio (ELR)	ELR = (FNR+PNR) /(FRR+PRR)	Total nonrenewable resource emergy flows to the total renewable resource emergy flows. The lower the ratio, the lower the pressure on the ecological environment.
Purchased input ratio (PIR)	PIR = (PRR+PNR)/(FRR+FNR)	Ratio of purchased resource emergy flows over the sum of free natural resource emergy input. The lower the ratio, the smaller the reliance on outsourced resources.
Self-sufficiency Ratio (SSR)	SSR = (FRR+FNR)/U	Represents the free natural resource inputs to total inputs.
Emergy yield ratio (EYR)	EYR = Y^em^/(PRR+PNR)	System yield (Y^em^) divided by the purchased resource emergy flow input.
Carbon-emergy output intensity (CemI)	CemI = CO_2-eq_ / emergy output	CO_2-eq_ emissions per unit yields measured in emergy.
Carbon-monetary output intensity (CmI)	CmI = CO_2-eq_ / monetary output value	CO_2-eq_ emissions per unit yield measured in monetary terms. All monetary value is converted to 1978 prices based on the price index.

Note: Data sets for this table please see Table C in S1 File.

### 2.2 Nonseparable undesirable output SBM DEA model

Traditional DEA is a nonparametric linear programming technique to study the relative efficiency scores of different decision-making units (DMUs) compared to the available best practice production model. Many studies have employed the DEA model in considering undesirable outputs for efficiency evaluation in the agricultural sector [14, 55, 56]. In the current study, we want to emphasize the inseparability of undesirable outputs. For example, CO_2_ emissions are nonseparable undesirable outputs caused by fossil fuel use, and N_2_O emissions are nonseparable undesirable outputs caused by using nitrogenous fertilizer inputs. Murty, Russell and Levkoff proposed a byproduction approach that makes a distinction between conventional technology and byproduction technology. This is also in line with the reasoning that the pollutant itself would be linked to pollution-generating inputs [57]. In the current study, inputs and undesirable (carbon emissions) and desirable (value added of crop plantation) outputs were evaluated using a nonseparable undesirable output SBM-DEA model [23, 24] to measure the efficiency and carbon reduction potential of crop production systems.

The nonseparable undesirable SBM-DEA model assumes a connection between desirable (good) and undesirable (bad) outputs. Tone [23], Tone and Tsutsui [24] separate the set of good and bad outputs (*Y*^*g*^, *Y*^*b*^) into (*Y*^*sg*^, *Y*^*sb*^) and (*Y*^*nsg*^, *Y*^*nsb*^), where s and ns, g and b denote the separable and nonseparable, good (desirable) and bad (undesirable) outputs, respectively. Inputs can be divided into (*X*^*s*^, *X*^*ns*^), denoting separable and nonseparable input data matrices, respectively. A reduction of the nonseparable inputs and bad (undesirable) outputs are designated by αx^ns^, αy^nsb^, where 0 ≤ α ≤ 1, assuming the same reduction index α for inputs and bad (undesirable) outputs. Accompanied by the nonseparable setting, a new production possibility set *P*_*ns*_ is defined by:

$$P_{ns}=\left\{\left(x^{s},x^{ns},Y^{sg},Y^{sb},y^{nsb}\right)|\begin{array}{c} x^{s}\geq X^{s}\lambda ,x^{ns}\geq X^{ns}\lambda ,y^{sg}\leq Y^{sg}\lambda ,y^{sb}\geq Y^{sb}\lambda ,\\ y^{nsb}\geq Y^{nsb}\lambda \end{array}\right\}$$ (1)

An SBM-DEA model with nonseparable inputs and outputs can be the following equation:

$$\rho^{*}=min\frac{1-\frac{1}{m}\sum_{i=1}^{m1}\frac{s_{i}^{s-}}{x_{i0}^{s}}-\frac{1}{m}\sum_{i=1}^{m2}\frac{s_{i}^{ns-}}{x_{i0}^{ns}}-\frac{m_{2}}{m}\left(1-\alpha \right)}{1+\frac{1}{s}\left(\sum_{r=1}^{s_{11}}\frac{s_{r}^{sg}}{y_{r0}^{\text{sg}}}+\sum_{r=1}^{s_{22}}\frac{s_{r}^{nsb}}{y_{r0}^{nsb}}+\left(s_{21}+s_{22}\right)\left(1-\alpha \right)\right)}$$ (2)

$$\text{Subject}\;\text{to}\;x_{0}^{s}=X^{s}\lambda +S^{s-};\alpha x_{0}^{ns}=X^{ns}\lambda +S^{ns-}$$

$$y_{0}^{sg}=Y^{sg}\lambda -S^{sg-};\alpha y_{0}^{nsg}\leq Y^{nsg}\lambda ;\alpha y_{0}^{nsb}=Y^{nsb}\lambda +S^{nsb}$$

$$\sum_{r=1}^{s_{11}}\left(y_{r0}^{sg}+s_{r}^{sg}\right)+\alpha \sum_{r=1}^{s_{21}}y_{r0}^{nsg}=\sum_{r=1}^{s_{11}}y_{r0}^{sg}+\sum_{r=1}^{s_{21}}y_{r0}^{nsg};\frac{S_{r}^{sg}}{y_{r0}^{sg}}\leq U\left(\forall r\right)$$

$$s^{s-}\geq 0,s^{ns-}\geq 0,s^{sg}\geq 0,s^{nsb}\geq 0,\lambda \geq 0,0\leq \alpha \leq 1.$$

where ρ* is the efficiency score, α is the reduction proportion of inputs or outputs, and U is the extendable upper limit of separable desirable outputs. s^s-^, s^ns-^, s^sg^ and s^nsb^ are slacks of separable inputs, nonseparable inputs, separable desirable outputs, and nonseparable undesirable outputs, respectively.

The objective function is strictly monotonically decreasing with respect to $s_{i}^{s-}\left(\forall i\right),s_{r}^{sg}\left(\forall r\right),$ and α. The DMU is efficient if and only if $\rho^{*}=1,i.e.,S^{s-*}=0,\alpha^{*}=1$.

If the DMU is inefficient, i.e., ρ*<1, it can be improved and become efficient using the following projection:

$$x_{o}^{s}⇐x_{o}^{s}-S^{s-*};x_{o}^{ns}⇐\alpha^{*}x_{o}^{ns};y_{0}^{sg}⇐y_{0}^{sg}+s^{sg*};y_{o}^{nsb}⇐\alpha^{*}xy_{o}^{nsb}$$

The decision-making units (DMUs) in the current research are the crop plantation system of the 11 prefectures or cities in Zhejiang Province. The four input variables, labor, sown land, nitrogen fertilizer and mechanical power, were selected as the most important affecting factors of agricultural productivity. Table 3 reports the descriptive statistics of the variables.

**Table 3:** Summary statistics of the DMUs’ input and output variables.

Variable	Unit	Mean	Max	Min	Std.Dev
Input:					
Labor input	10^4^ persons	61.73	129.68	4.63	33.97
Sown land	million ha.	282.90	512.10	13.45	118.19
Nitrogen fertilizer	10^4^ tons	5.17	10.35	0.24	2.41
Mechanical power	million kW	192.39	360.00	51.10	75.32
Output:					
Value added of crop plantation	100 million RMB	51.79	168.57	1.78	37.86
CO_2-eq_ emission	10^4^ tons	160.94	268.54	14.06	61.66

Labor was measured by the “Employment in crop planting”. Employment is defined as the number of people of working age engaged in agricultural crop planting activities. Sown land was measured by the extension (in thousand ha) of the sown land. Mechanical power was used as a proxy for fuel and energy inputs. Nitrogenous fertilizer consumption was measured in fertilizer purity (in tons). Finally, the output was expressed by the crop plantation value added. The CO_2-eq_ emission was accounted for according to the IPCC method [41].

In the nonseparable undesirable SBM-DEA model, the sown area and labor force were treated as separable inputs, and energy consumption and nitrogen fertilizer were treated as nonseparable inputs. The value added of crop plantation was treated as the desirable output and CO_2-eq_ emission as a nonseparable undesirable output. Since CO_2_ emission is a byproduct of energy consumption, CO_2_ emission and energy consumption were treated as nonseparable variables. N_2_O emissions and nitrogen fertilizer were treated as nonseparable variables. The estimation of efficiency scores through nonseparable output SBM-DEA models was conducted using the MaxDEA software.

All the input and output data of crop production were drawn from local statistical year books in the 11 prefectures and Zhejiang Province [44–54]. Some missing data were estimated through cross-referencing and proportional calculation (see Table D in S1 File).

### 2.3 Analytical boundary and framework

**Fig 1:**
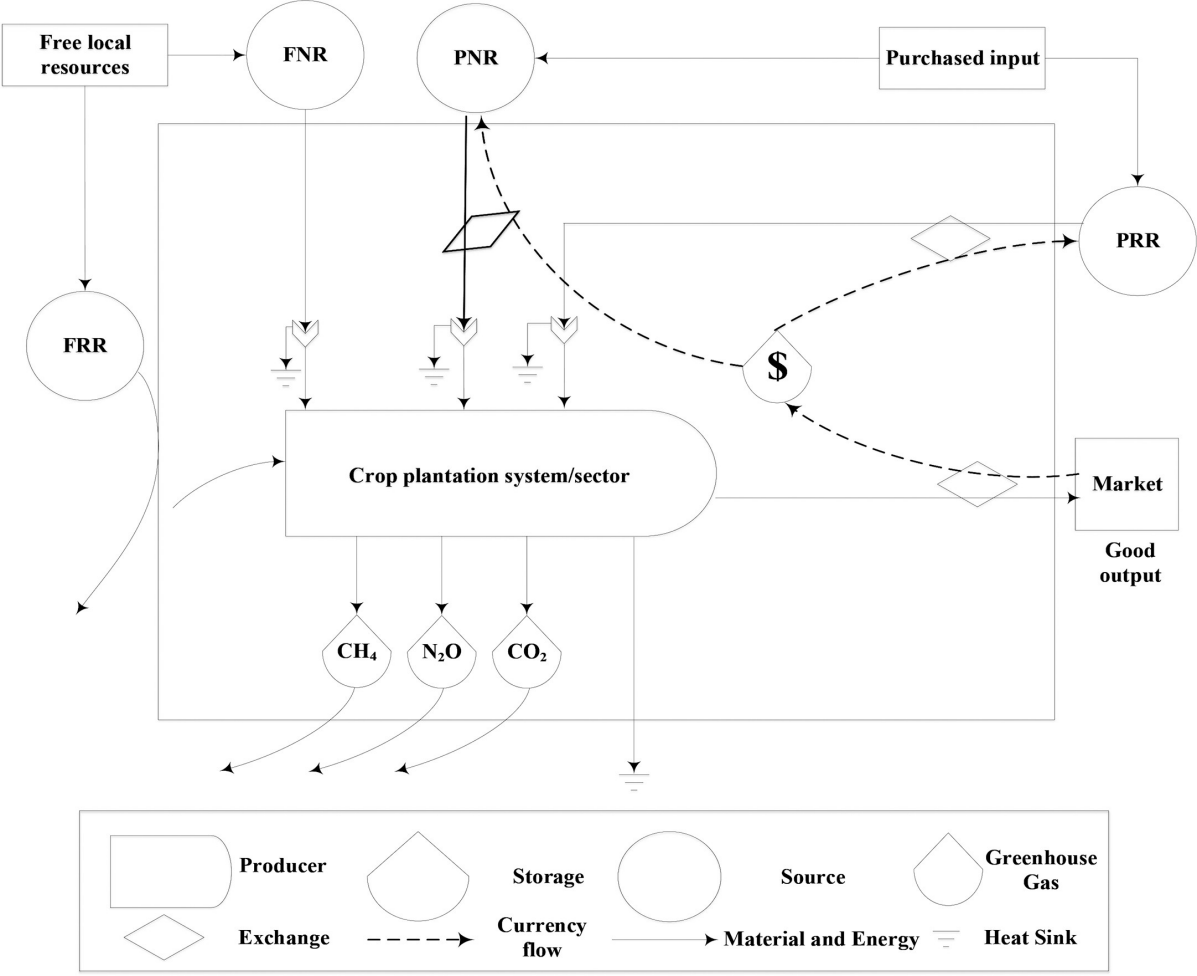
Diagram of the boundary of crop plantation system of the current study.

**Fig 1** illustrates the analytical boundary and framework of the emergy and emission accounting and DEA analysis of the crop plantation systems in the current study.

Within the emergy and emission accounting and DEA analysis framework, FRR and FNR, such as sunlight, rainfall and topsoil, are attributed to sown land area input. PNR includes inputs such as machinery, diesel fuel, electrical power and nitrogen fertilizer, which the direct and indirect undesirable outputs, CO_2_ and N_2_O emissions, respectively, are associated with.

## 3. Results

### 3.1 Emergy accounting for resource input and output

#### 3.1.1 Various resource input changes in crop production

**Fig 2:**
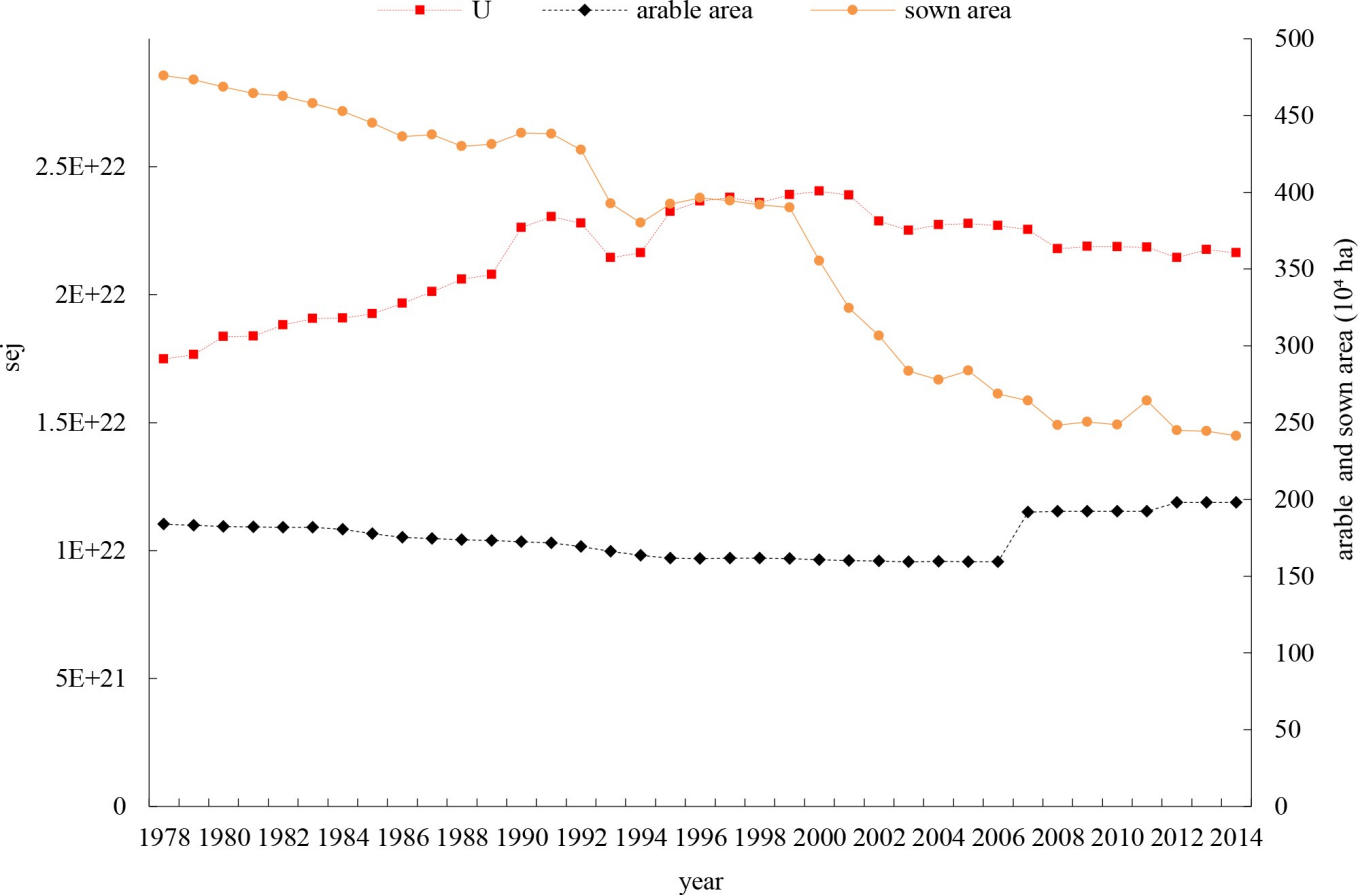
Change trend of total input (U) and arable and sown area during 1978–2014.

**Fig 2** shows that total resource inputs measured in emergy (U = FRR+FNR+PRR+PNR) in crop production systems increased from 1978 to 2001 and then began decreasing slightly after 2002. Total input (U) in 2014 (2.16E22 sej) increased by 23.72% compared with 1978 (1.75E22 sej). Arable area showed a deceasing trend from 1978 to 2006 and then slightly increased subsequently. The sown area declined sharply.

**Fig 3:**
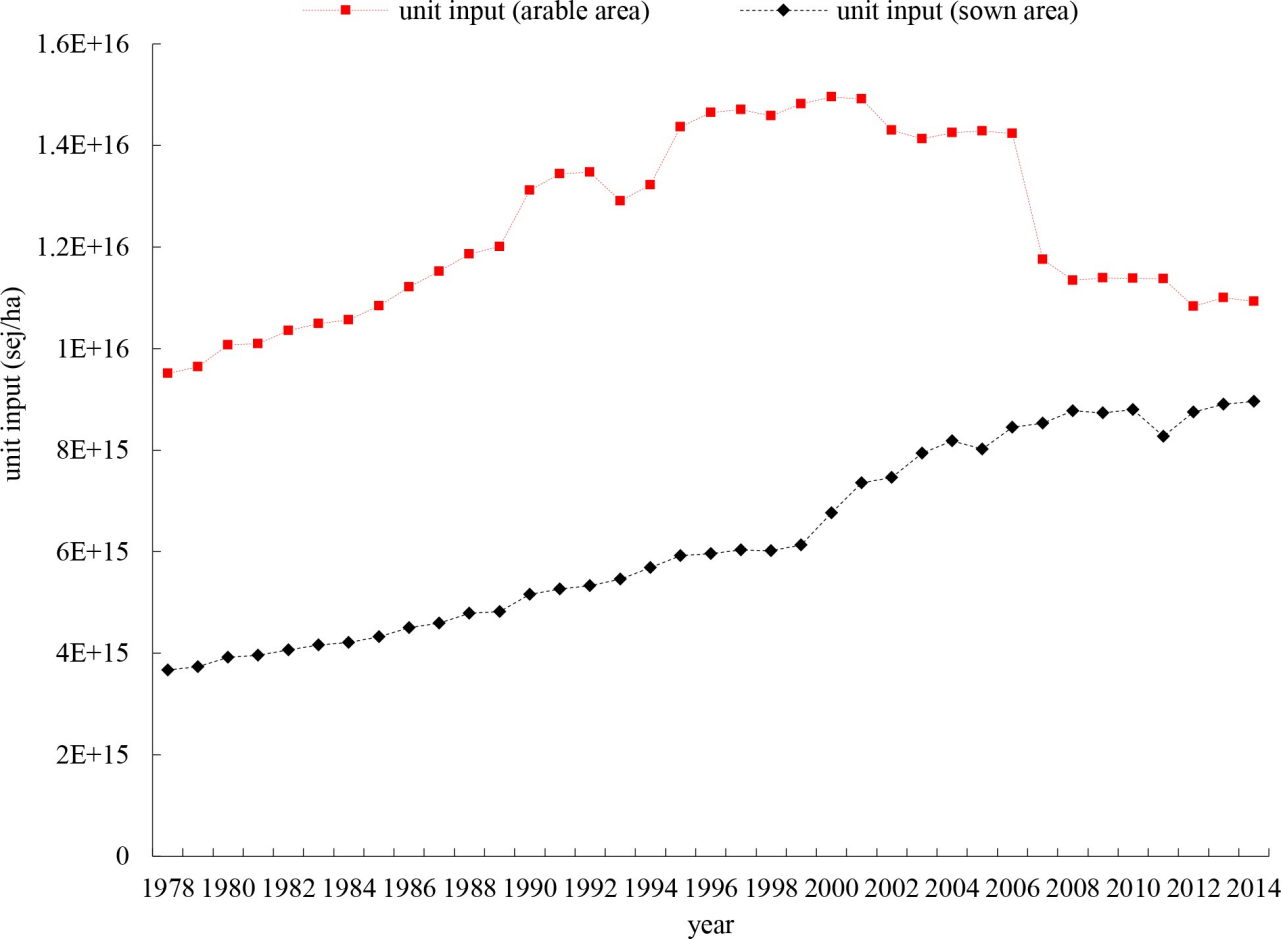
Annual emergy input density (EID) measured by emergy input per unit arable and sown area (sej/ha.) in crop production in Zhejiang province.

**Fig 3** shows the trend of annual emergy input densities (EIDs) measured by emergy input per unit sown area and arable land area, respectively. The former has an upward trend during the study period, which shows an increasing dependence on resource inputs. The latter has an increasing trend from 1978 to 2006 and then deceases due to the increase of arable area.

**Fig 4:**
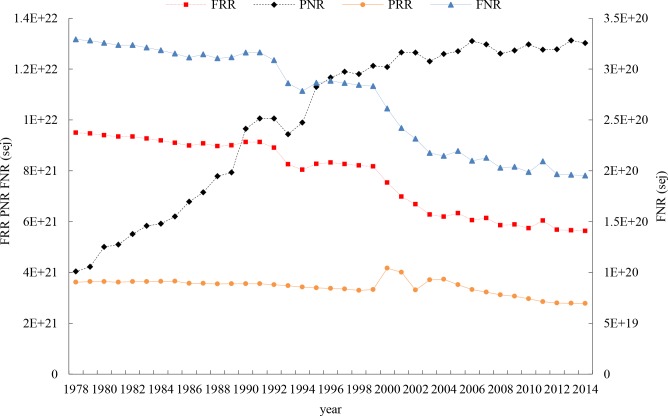
Yearly change of emergy inputs by categories in crop production in Zhejiang province.

**Fig 4** illustrates a significant change in various agricultural emergy inputs. During the study period, FRR (including sown land area associated rainfall and sunshine), FNR (representing sown land area associated topsoil), and PRR (labor and irrigation water) declined by 40.71%, 40.71% and 23.09%, respectively, because of the continuous decrease in sown area over the past 30 years. Meanwhile, PNR (chemicals and energy) increased by 222.29%, from 4.04E21 sej in 1978 to 1.30E22 in 2014. The decline of inputs of renewable natural resources (FRR+PRR) and an overdependence on purchased nonrenewable resources (PNR) suggests there is an unsustainable trend of the crop production model in Zhejiang province.

#### 3.1.2 Measuring unsustainability

The EID indicator alone cannot measure the level of unsustainability of crop production. The following indicators assist in this case: the environmental load indicator (ELR), the purchased input ratio (PIR) and the self-sufficiency ratio (SSR).

**Fig 5:**
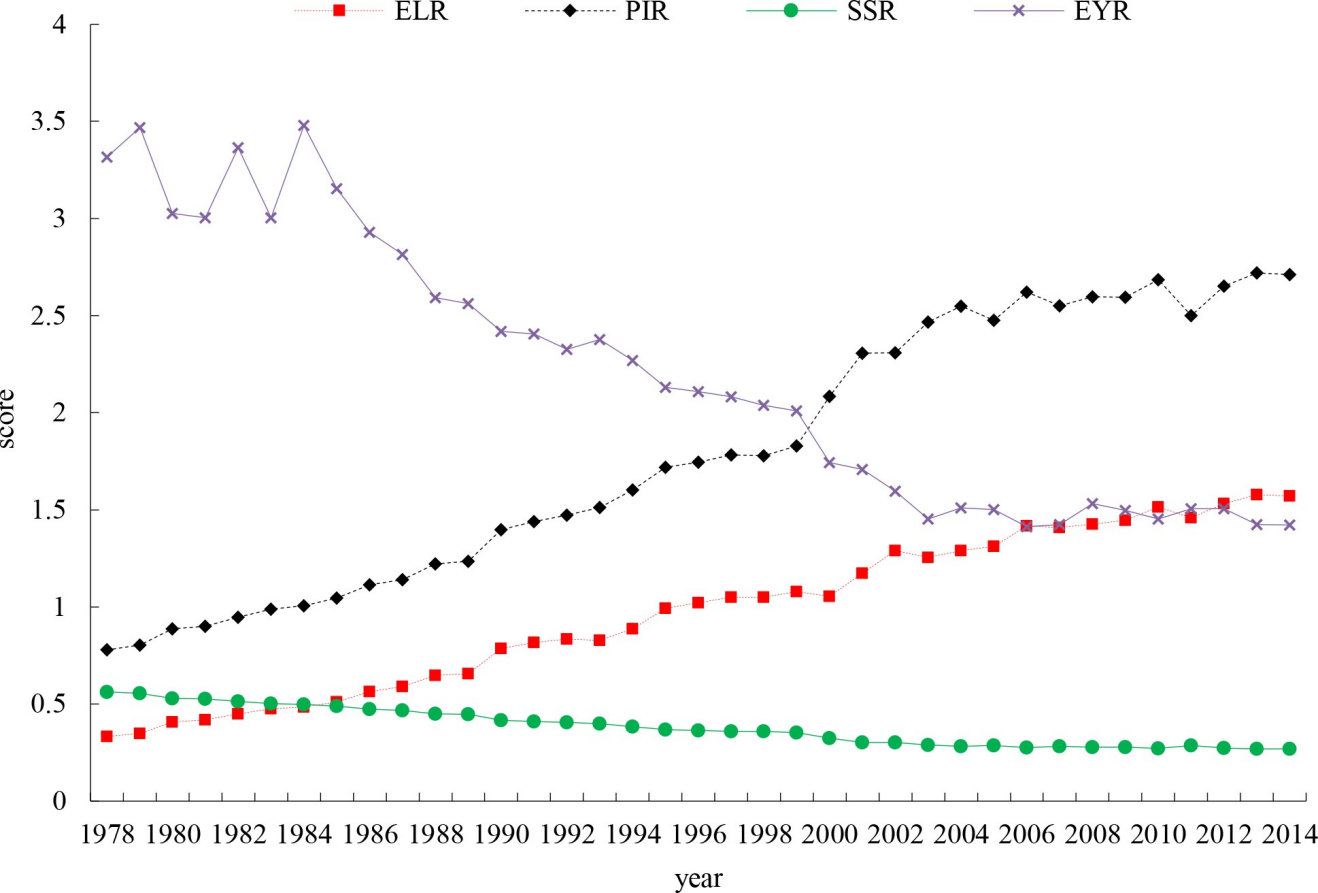
ELR, PIR, SSR and EYR indices of Zhejiang crop production systems.

The environmental load indicator (ELR = (FNR+PNR)/(FRR+PRR)), or the ratio of nonrenewable inputs to renewable resource factor inputs, experienced an upward trend, indicating an increasing environmental load of agricultural production due to an overdependence on nonrenewable resources to maintain crop production. The ratio of purchased resources to free resource factors (PIR = (PRR+PNR)/ (FRR+FNR)) also increased. The higher the ratio, the more reliance on purchased inputs compared to nonpurchased inputs. The self-sufficiency ratio (SSR = (FRR+FNR)/U) indicator exhibited a decreasing trend for the ratio of renewable inputs to total inputs. The changes of the ELR, PIR and SSR are shown in **Fig 5**.

The emergy yield indicator (EYR = Y/ (PRR+PNR)) decreased during the study period, indicating a decreasing payback rate for purchased inputs (PRR+PNR).

#### 3.1.3 Structural changes of purchased nonrenewable inputs

During the study period, PNR sharply increased and eventually outweighed the other three categories of inputs (**Fig 4)**, and it was the main source of CO_2_ and N_2_O emissions. Thus, a closer observation of the change in the formation of PNR is necessary.

**Fig 6:**
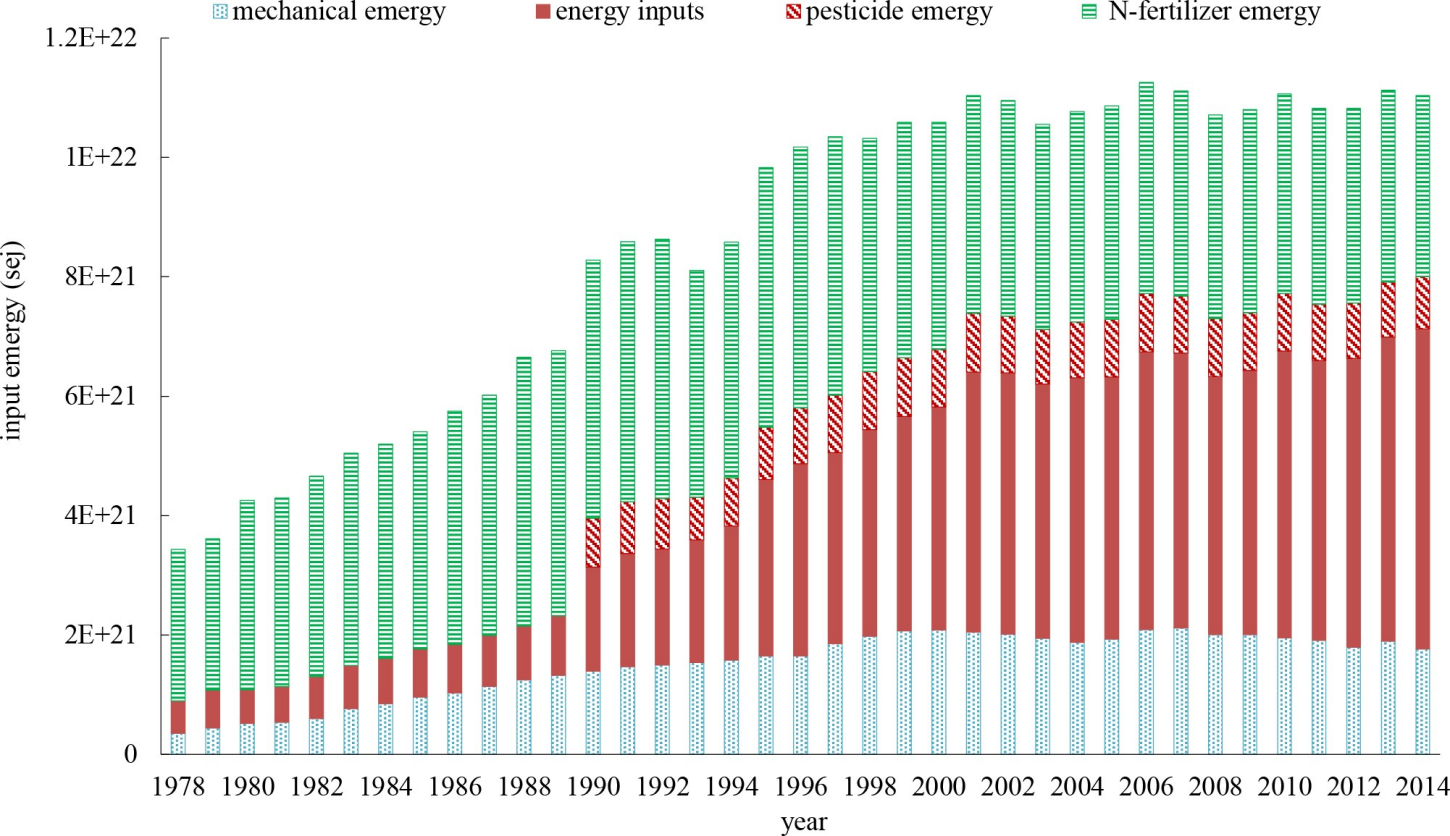
Purchased nonrenewable inputs (PNR) component structure in terms of emergy.

As illustrated by the time series of emergy flows of the PNR components (**Fig 6**), chemical nitrogen fertilizer (N-fertilizer) and energy inputs were the main purchased nonrenewable resources. The amount of N-fertilizer use initially rose and then fell during the period of 1978–2014. The increase of N-fertilizer use was due to the implementation of a household responsibility system (HRS), which encouraged farmers to increase inputs and raise crop yields. Its subsequent decrease was due to the decline of sown area, the application of balanced fertilization technology, and the promotion of organic fertilizer use. The fossil fuels emergy flow increased almost 10-fold from 1978 to 2014. The emergy flow proportion of mechanical equipment increased from 8.74% in 1978 to 13.56% in 2014. Pesticide emergy input levels were relatively stable from 1990 onward.

### 3.2 Low-carbon illusion based on CmI accounting and the high-carbon reality based on CemI accounting

#### 3.2.1 Carbon accounting of crop production systems

GHG emissions embodied in crop plantations were primarily from rice paddies (CH_4_ emission) and PNR inputs. The calculation of carbon emissions embodied in PNR inputs was based on the LCA method proposed by ISO 14000 [41, 42, 58]. Total GHG emissions peaked in 1990 at 21.07 million tons of CO_2-eq_. From 1978–1986, rice cultivation-induced CH_4_ accounted for approximately 50% of total GHG emissions. Its proportion then declined to 20% in 2014 with a decrease in rice paddy area.

**Fig 7:**
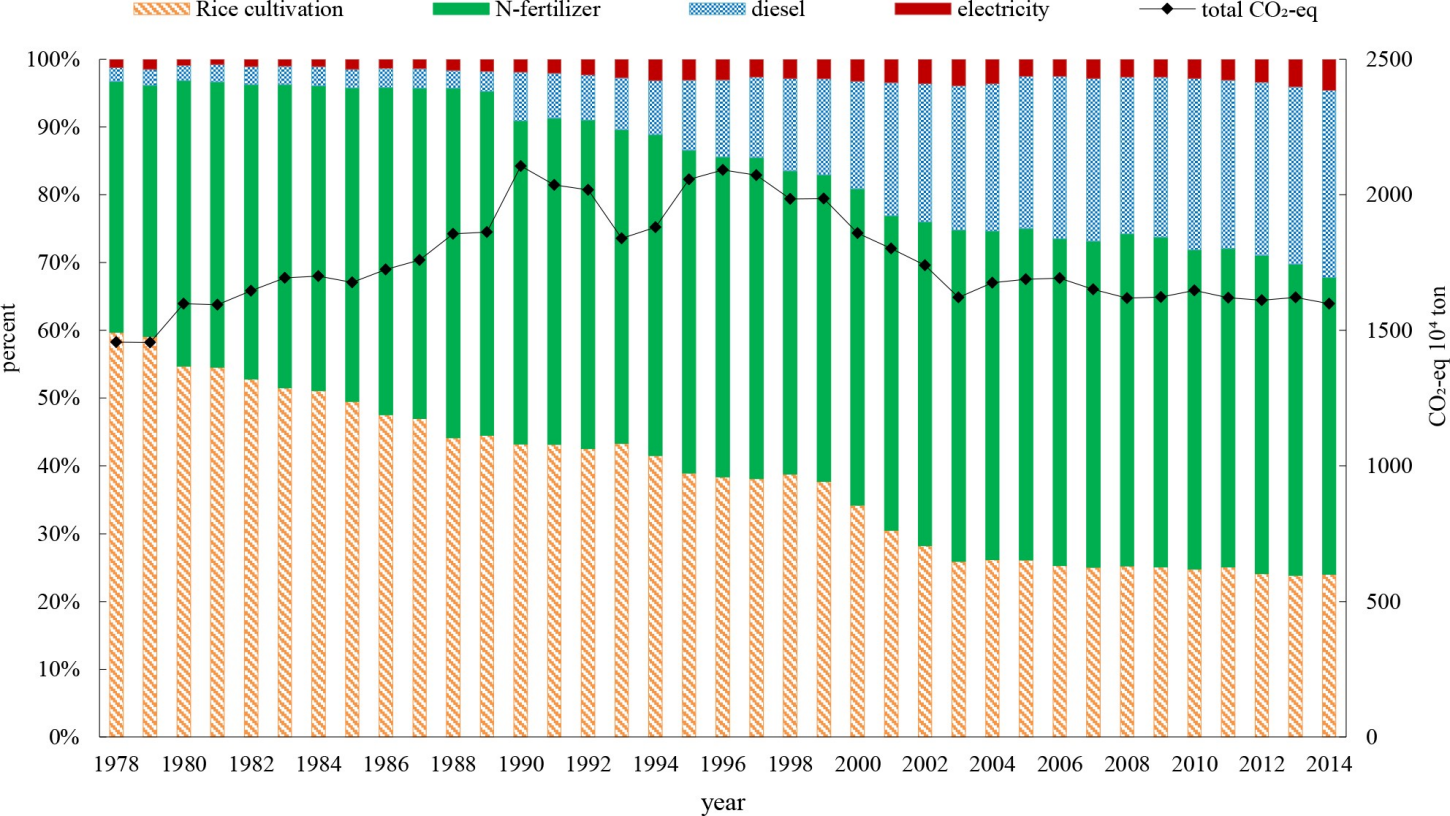
GHG emission structure of the crop plantation sector in Zhejiang province.

As shown in **Fig 7**, carbon emissions embodied in N-fertilizer applications have always been a large part of total emissions, ranging from 36.88% in 1978 to 51.33% in 1988. In 2014, it was 40.22%. Carbon emissions embodied in diesel production rose sharply to exceed rice paddy emissions and became the second largest emissions source in 2010. These changes were due to more extensive use of agricultural machinery in recent years.

#### 3.2.2 Conflicting carbon intensity tendencies based on CmI and CemI accountings

**Fig 8:**
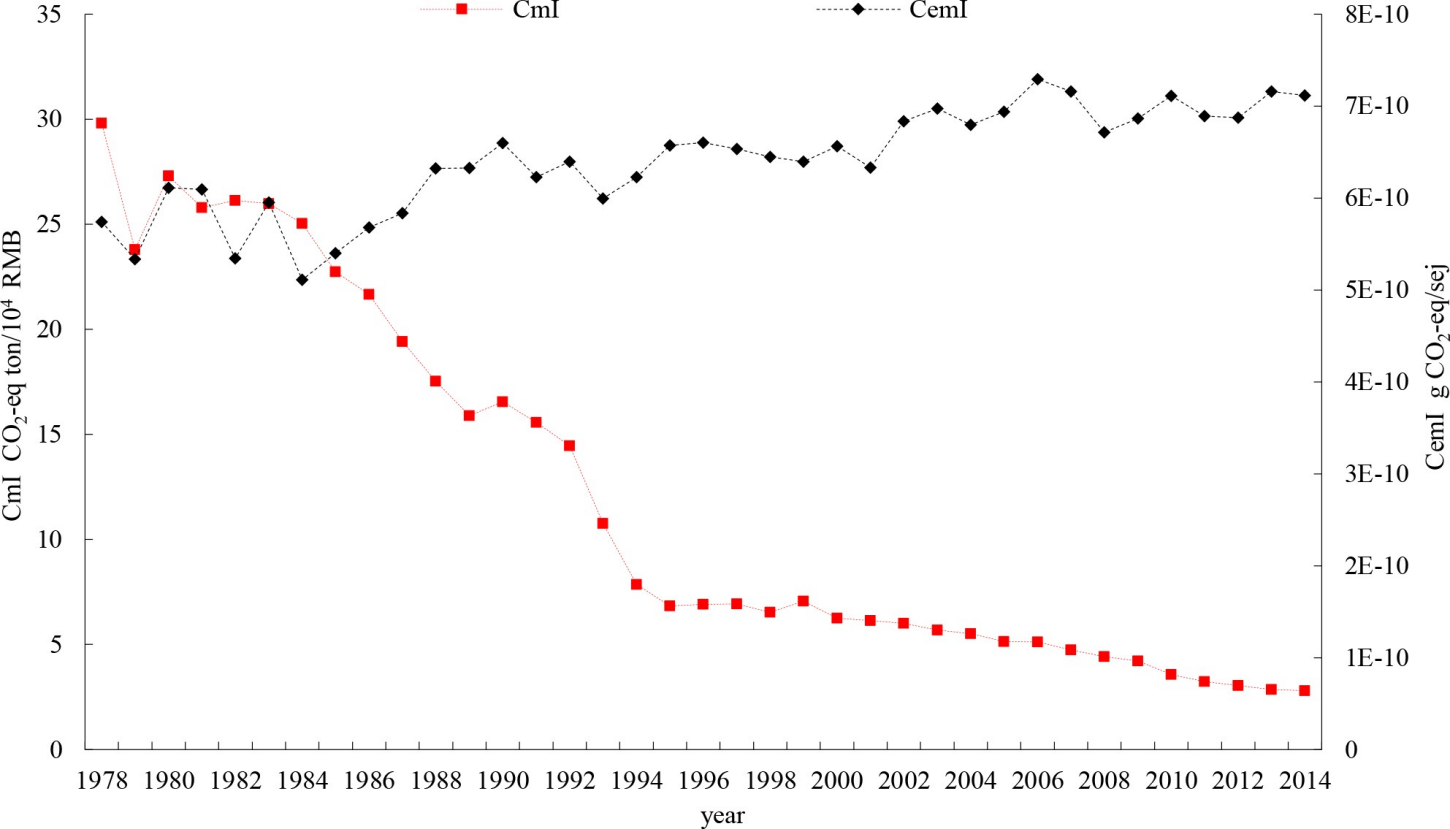
Carbon-monetary output intensity (CmI) and Carbon-emergy output intensity (CemI) in Zhejiang province.

Carbon intensity measured in monetary output (CmI) declined from 29.82 tons CO_2-eq_/ten thousand RMB in 1978 to 2.81 tons CO_2-eq_/ten thousand RMB in 2014, with a sharp decreasing rate of 90.58% (**Fig 8**). However, the indicator of carbon-emergy output intensity (CemI) increased 23.98% from 5.74 g CO_2-eq_ /10^10^ sej in 1978 to 7.12 g CO_2-eq_ /10^10^ sej in 2014.

Although the CmI has decreased, the fact that the CemI indicator has increased indicates an increasing carbon intensity trend in the crop farming sector in Zhejiang province. Thus, the decline in carbon intensity based on the monetary output indicator conflicts with the increasing carbon intensity indicator that is based on the emergy indicator.

**Fig 9:**
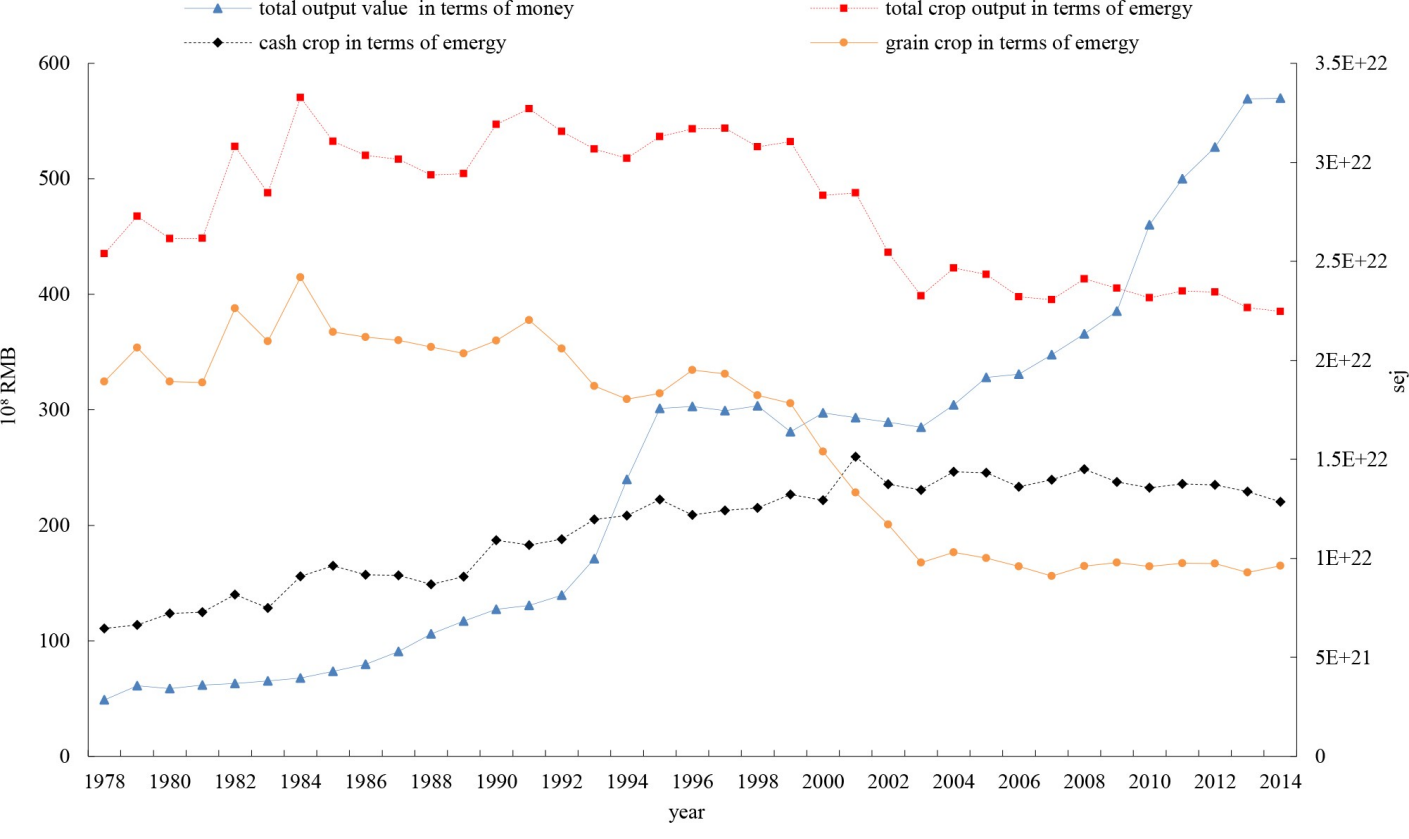
Crop outputs measured in terms of emergy and monetary value in Zhejiang province.

From **Fig 9**, CmI and CemI experienced very similar time series shapes from 1978–1984. This is due to the consistent crop plantation structure. After 1984, the carbon intensity measured by per unit monetary output value continuously decreased. However, this is just a ‘low carbon illusion,’ which was not caused by a carbon reduction in crop plantations (according to **Fig 7**, total carbon emissions embodied in crop plantations have not been decreasing sharply) but was instead caused by an increase of output measured in monetary terms due to the expansion of cash crops (and at the same time a decrease of grain crops).

Within crop plantations, in addition to grain crops, there are higher value crops (or cash crops), including economic crops (oil, cotton, sugar, tobacco, etc.) and horticultural crops (vegetables, fruits, tea, etc.). Due to the decrease in the sown area of grain crops, along with the increase of cash crops, since 2001, the output of cash crops (measured by emergy) has surpassed that of grain crops. From 1978–2014, although the total output measured by emergy first increased and then decreased (due to total sown land area shrinkage), a dramatic increase in the total output measured in terms of monetary value (output value) can be seen (see **Fig 9**). This produced the divergence in the change trends of carbon intensities indicated by carbon-money output intensity (CmI) and carbon-emergy output intensity (CemI).

The increase of cash crops (and the simultaneous decrease of grain crops) was also responsible for the increases in the use of PNR and the emergy input per unit sown area because cash crop plantations are generally more chemical and energy intensive than grain crop plantations.

### 3.3 Mitigation potential based on DEA analysis

In the previous section, emergy input and output indicators and carbon emissions intensity accounting were applied to describe the carbon efficiency of crop production. Specifically, the emergy-based carbon efficiency (CemI) indicated that a low efficiency problem in crop plantation systems may exist. However, CemI alone does not give an indication of a system’s carbon reduction potential. To determine the reduction potential, the nonseparable undesirable output SBM-DEA method was employed to reveal the input redundancy and the undesirable outputs (GHG emissions) of crop production for each prefecture in the province.

**Fig 10:**
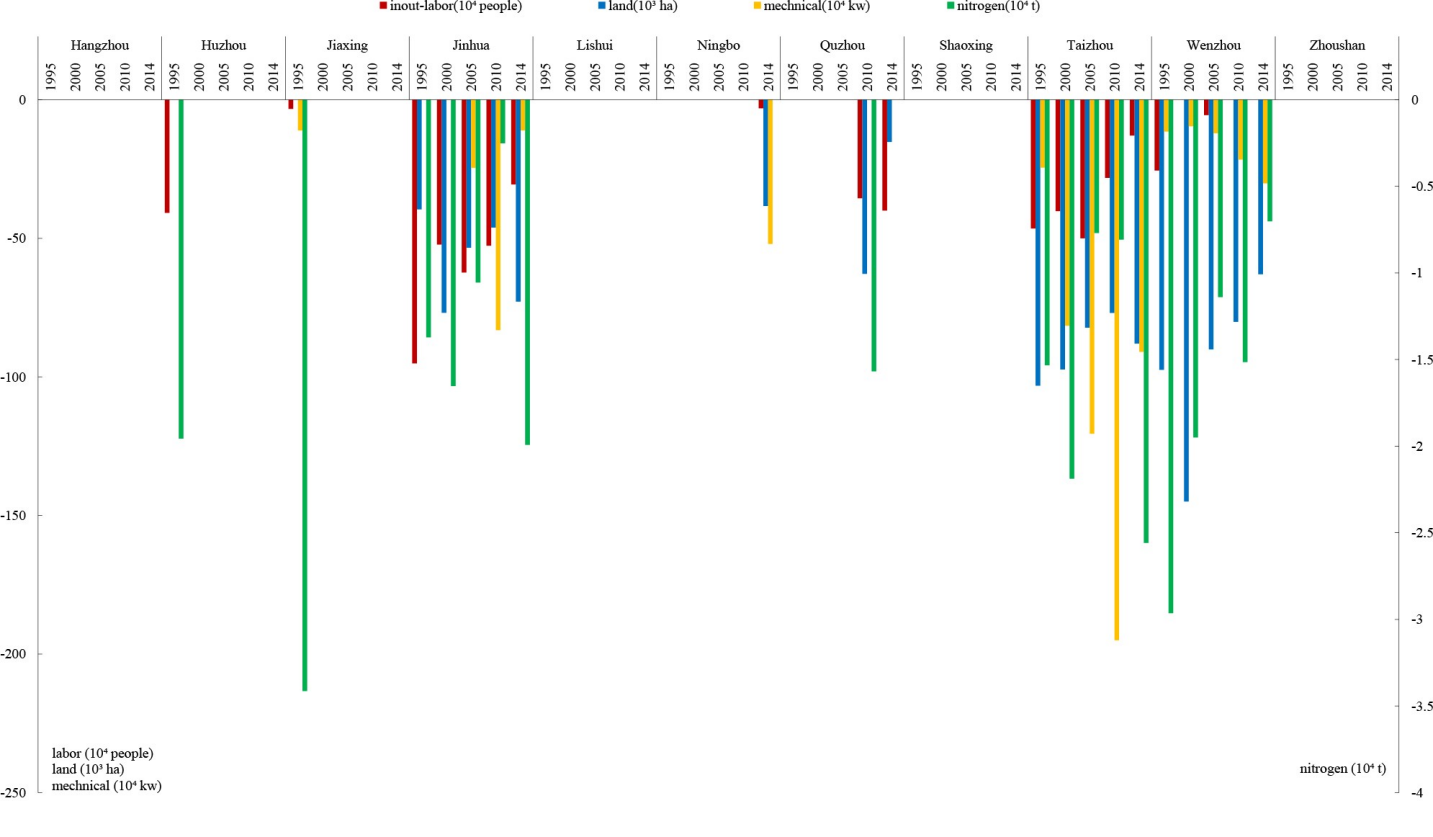
Input factors redundancy estimation for the prefectures of Zhejiang province.

Due to data availability, only the production efficiency value (**Table 4**), input factors redundancy estimation (**Fig 10**) and the associated carbon mitigation potential (**Table 5**) were estimated for the years 1995, 2000, 2005, 2010 and 2014. The results are listed in Table 4.

**Table 4:** Crop production efficiency value of various prefectures of Zhejiang Province in selected years.

Region	1995	2000	2005	2010	2014	average
Hangzhou	1	1	1	1	1	1
Huzhou	0.8128	1	1	1	1	0.9626
Jiaxing	0.8853	1	1	1	1	0.9771
Jinhua	0.7074	0.70244	0.64770	0.6429	0.6042	0.6609
Lishui	1	1	1	1	1	1
Ningbo	1	1	1	1	0.8515	0.9703
Quzhou	1	1	1	0.6299	0.8199	0.8899
Shaoxing	1	1	1	1	1	1
Taizhou	0.6684	0.6020	0.5631	0.5514	0.5139	0.5798
Wenzhou	0.6184	0.6535	0.6905	0.7142	0.7543	0.6862
Zhoushan	1	1	1	1	1	1

**Table 5:** Carbon mitigation potential based on emergy output in the crop production systems of various prefectures of Zhejiang province in selected years.

Year	1995	2000	2005	2010	2014	Total
Carbon mitigation potential Unit	10^4^ tons CO_2-eq_					
Hangzhou	0	0	0	0	0	0
Huzhou	0	0	0	0	0	0
Jiaxing	0	0	0	0	0	0
Jinhua	20.77	40.22	31.33	27	46.19	165.50
Lishui	0	0	0	0	0	0
Ningbo	0	0	0	0	24.76	24.76
Quzhou	0	0	0	36.78	9.25	46.03
Shaoxing	0	0	0	0	0	0
Taizhou	54.11	50.88	48.19	44.97	55.56	253.70
Wenzhou	51.11	75.85	52.82	46.88	39.77	266.43
Zhoushan	0	0	0	0	0	0
Total	125.99	166.94	132.34	155.63	175.52	-

Table 4 presents the results of the production efficiency value of various prefectures of the province. Each prefecture in a specific year was treated as a separate individual entity (i.e., the same prefecture observed in two different years was treated as two separate entities). The table shows that, of the 5 selected years, 35 of the total 55 observations are fully efficient. This is very high proportion and can be caused by a relatively small number of observations within the same province. However, the results did uncover inefficiencies in some prefectures, which is consistent with the development level and geographical characteristics of the prefectures in Zhejiang province, so the number of observations is not 'too small'.

**Tables 4 and 5 and Fig 10** indicate that Jinhua, Taizhou, and Wenzhou, which are primarily located in the southern part of the province, exhibit relatively lower production efficiencies, higher input factor redundancies (especially machinery and chemical fertilizer use which are carbon intensive), and higher carbon emissions reduction potentials for crop production. Although the northern prefectures, such as Hangzhou, Huzhou, and Jiaxing, have relatively higher production efficiencies, they have lower input factor redundancy and less carbon reduction potential.

The annual total mitigation of emissions through the overall study period shows a slight increasing trend, which corresponds to the high carbon trend revealed by the CemI calculation.

## 4. Discussion and conclusion

This study applied a thermodynamics indicator, emergy, to account for the quantity of different types of inputs in crop production—namely, free renewable natural resources (FRR), free nonrenewable natural resources (FNR), purchased renewable resources (PRR) and purchased nonrenewable resources (PNR)—and the total crop production (Y) to be able to compare and analyze these factors. GHG emissions embodied in crop production are accounted for to show the carbon intensity of crop production, in terms of per unit emergy and monetary output, during the study period. A nonseparable undesirable output SBM-DEA analysis was used to reveal the production efficiency variation and the carbon emission mitigation potential of crop farming in Zhejiang province.

The emergy input density (EID) showed an increasing trend of emergy input per unit area, implying an increasing dependence on resource inputs. The ecological environmental loading radio (ELR) experienced an upward trend due to an overdependence on nonrenewable resources used to maintain crop production. The purchased input ratio (PIR) also continued to increase, thus implying increasing reliance on purchased inputs. The self-sufficiency ratio (SSR) presented a decreasing trend of the share of renewable inputs in input totals. These four indicators indicated that sustainability was not optimum in the province’s crop production.

GHG emissions from crop plantation were primarily related to PNR inputs based on the LCA method. Carbon intensity based on monetary output (CmI) declined. However, this does not mean that there are decreasing carbon emissions per unit of crop farming output. Cash crop plantations tend to produce a higher monetary value, which leads to an underestimation of actual carbon intensity per unit output. The carbon intensity indicator based on emergy (CemI) indicates an increasing carbon intensity trend in the crop farming sector, which reflects a more probable situation. This high carbon intensity trend is largely attributed to mechanization and the shift from labor-intensive to energy-intensive farming models.

Chemical fertilizers and energy utilization are the major GHG emissions sources. From the overall technical efficiency analysis based on the nonseparable undesirable output SBM-DEA, we inferred that an optimization of agricultural input allocation and, more specifically, reducing the use of nonrenewable resource inputs, such as chemicals and fossil fuels, would help to reduce GHG emissions.

The efficiencies in the areas of the northern prefectures of the province, namely, the Hangjiahu (Hangzhou, Jiaxing, Huzhou) and the Ningshao plains (Ningbo, Shaoxing), are higher than those of the southern areas, especially in the Jinqu basin (Jinhua, Quzhou) and other areas in the middle hilly region.

Currently, to keep agricultural production profitable, shifting to cash crop farming and increasing the use of outsourced, nonrenewable resource inputs with high direct and embodied carbon emissions has become a “must” in both Zhejiang Province and the nation.

The governmental departments of both Zhejiang Province and China have realized the urgency in promoting low carbon agriculture, and a series of actions are being implemented. Zhejiang province’s government has drawn up the "Zhejiang Modern Ecological Circular Agriculture Development Plan" [59]. In this plan, both an increase in chemical fertilizer use efficiency and a reduction in chemical fertilizer use in crop plantation is required. Technologies such as testing soil formula fertilization, organic nutrients (such as commercial organic fertilizers, biogas slurry, straw returning to the fields and green manure) and the replacement of chemical fertilizer are strongly encouraged. Controlling methane emissions is also addressed through water and fertilizer management improvement.

A green subsidy to support these reforms and technical progress is underway. Due to the small-scale nature of the average Chinese farmer, low carbon agriculture reform generally lacks investment for R&D. Therefore, financial support for low carbon agriculture technology development and expansion becomes a priority for policymakers in China. During 2011–2015, the Zhejiang Provincial government arranged 90 million RMB annually to support eco-circular agriculture development in the province, and the budget has been increased to 190 million RMB for 2016–2020. Though the subsidy is still small, compared with the large scale of production, it demonstrates substantial progress. The annual subsidy from the central government for testing soil formula fertilization have amounted to 700 million RMB since 2015. In 2013, Zhejiang Province regulated subsidies to encourage the phasing out of high-energy-consumption agricultural machinery [60].

The cancellation of subsidies to high carbon production factors, especially chemical fertilizer production and consumption, is another major countermeasure [61]. In 2015, the Ministry of Agriculture of China set the target of “zero increase of agricultural chemical fertilizer application by the year of 2020”. In response to the national target, the subsidies to chemical fertilizer production and sales have been cancelled, including the cancellation of preferential power and natural gas prices for chemical fertilizer production, a cancellation of the chemical fertilizer purchase subsidy, and the recovery of a value added tax on chemical fertilizer.

A low carbon agriculture promotion policy toolkit is expected to have profound effects in the near future.

## Supporting information

S1 File Table A. Variable correlation coefficient matrix.
Table B. Data sets of input and output based on emergy and carbon accounting.
Table C. Emergy and carbon emissions-based production efficiency indicators.
Table D. Inputs and outputs data in DEA analysis.
(DOCX)
