# Supplementary material for: Production efficiency and GHG emissions reduction potential evaluation in the crop production system based on emergy synthesis and nonseparable undesirable output DEA: A case study in Zhejiang Province, China
Source: PLoS One. 2018 Nov 1;13(11):e0206680. doi: 10.1371/journal.pone.0206680 (PMC6211734; doi:10.1371/journal.pone.0206680)
Supplement: S1 File — Table A. Variable correlation coefficient matrix. Table B. Data sets of input and output based on emergy and carbon accounting. Table C. Emergy and carbon emissions-based production efficiency indicators. Table D. Inputs and outputs data in DEA analysis. (DOCX) [file pone.0206680.s001.docx]

**Supporting Information**

**Table A in S1 File** Variable correlation coefficient matrix

| Variable | Labor force | Sown land | Nitrogen fertilizer | [Mechanical](javascript:;) [power](javascript:;) |
| --- | --- | --- | --- | --- |
| Labor force | 1 |  |  |  |
|  |  |  |  |  |
| Sown land | 0.2032 | 1 |  |  |
|  | (0.1368) |  |  |  |
| Nitrogen fertilizer | 0.016 | 0.9164 | 1 |  |
|  | (0.9076) | (0) |  |  |
| [Mechanical](javascript:;) [power](javascript:;) | -0.1525 | 0.2698 | 0.2995 | 1 |
|  | (0.2663) | （0.0464*） | （0.0263*） |  |

Note: * means p<5%

**Table B in S1 File** Data sets of input and output based on emergy and carbon accounting.

| year | Input resources (sej) | | | | Desirable crop outputs (sej) | | | | | | | | | | | | | | | | Carbon emissions  (total CO₂-eq, 10^4^ton) |
| --- | --- | --- | --- | --- | --- | --- | --- | --- | --- | --- | --- | --- | --- | --- | --- | --- | --- | --- | --- | --- | --- |
|  | FRR | FNR | PNR | PRR | wheat | rice | corn | potato | soybean | peanut | rapeseed | gingili | cotton | fiber crops | sugarcane | tobacco | vegetable | melon | silkworm | tea |  |
| 1978 | 9.49862E+21 | 3.29131E+20 | 4.04104E+21 | 3.61356E+21 | 2.592E+21 | 1.502E+22 | 8.624E+20 | 4.434E+20 | 1.2E+21 | 3.375E+19 | 8.153E+20 | 6.6E+18 | 1.965E+21 | 7.507E+19 | 2.112E+20 | 8.729E+18 | 4.512E+19 | 1.277E+20 | 1.941E+21 | 3.275E+19 | 1456.905 |
| 1979 | 9.46511E+21 | 3.2797E+20 | 4.22409E+21 | 3.63793E+21 | 3.002E+21 | 1.642E+22 | 8.162E+20 | 3.972E+20 | 1.2E+21 | 3.682E+19 | 1E+21 | 1.32E+19 | 1.811E+21 | 6.44E+19 | 2.187E+20 | 4.365E+18 | 4.512E+19 | 1.962E+20 | 2.006E+21 | 3.655E+19 | 1455.254 |
| 1980 | 9.40011E+21 | 3.25717E+20 | 5.00307E+21 | 3.63678E+21 | 3.135E+21 | 1.484E+22 | 4.774E+20 | 4.804E+20 | 1.2E+21 | 3.988E+19 | 1.071E+21 | 9.9E+18 | 2.244E+21 | 6.194E+19 | 1.934E+20 | 8.729E+18 | 4.512E+19 | 1.967E+20 | 2.103E+21 | 4.207E+19 | 1597.879 |
| 1981 | 9.34209E+21 | 3.23707E+20 | 5.09374E+21 | 3.61892E+21 | 3.046E+21 | 1.49E+22 | 4.928E+20 | 4.465E+20 | 1.2E+21 | 3.988E+19 | 1.508E+21 | 1.32E+19 | 1.844E+21 | 7.302E+19 | 1.845E+20 | 1.091E+19 | 4.512E+19 | 1.992E+20 | 2.113E+21 | 4.983E+19 | 1594.174 |
| 1982 | 9.34128E+21 | 3.23679E+20 | 5.50988E+21 | 3.64189E+21 | 3.609E+21 | 1.796E+22 | 5.698E+20 | 4.835E+20 | 1.2E+21 | 3.375E+19 | 1.461E+21 | 1.32E+19 | 2.642E+21 | 7.548E+19 | 2.299E+20 | 1.528E+19 | 4.512E+19 | 2.255E+20 | 2.171E+21 | 5.976E+19 | 1646.358 |
| 1983 | 9.26925E+21 | 3.21183E+20 | 5.83021E+21 | 3.64258E+21 | 3.6E+21 | 1.646E+22 | 4.158E+20 | 4.866E+20 | 1.2E+21 | 2.148E+19 | 1.134E+21 | 6.6E+18 | 2.537E+21 | 7.261E+19 | 1.598E+20 | 8.729E+18 | 4.512E+19 | 2.473E+20 | 2.009E+21 | 5.692E+19 | 1693.669 |
| 1984 | 9.19277E+21 | 3.18533E+20 | 5.91769E+21 | 3.64891E+21 | 4.222E+21 | 1.905E+22 | 4.466E+20 | 4.681E+20 | 1.2E+21 | 2.761E+19 | 1.288E+21 | 9.9E+18 | 3.598E+21 | 8.409E+19 | 2.325E+20 | 8.729E+18 | 4.512E+19 | 2.683E+20 | 2.27E+21 | 5.334E+19 | 1700.405 |
| 1985 | 9.09643E+21 | 3.15194E+20 | 6.19576E+21 | 3.65267E+21 | 3.477E+21 | 1.713E+22 | 4.066E+20 | 4.225E+20 | 1.2E+21 | 3.375E+19 | 1.678E+21 | 1.65E+19 | 2.201E+21 | 1.03E+20 | 3.618E+20 | 1.091E+19 | 4.512E+19 | 1.183E+21 | 2.737E+21 | 5.195E+19 | 1677.306 |
| 1986 | 8.9886E+21 | 3.11458E+20 | 6.78398E+21 | 3.57344E+21 | 3.191E+21 | 1.726E+22 | 3.419E+20 | 3.708E+20 | 1.2E+21 | 3.682E+19 | 1.627E+21 | 1.32E+19 | 2.047E+21 | 8.204E+19 | 4.348E+20 | 8.729E+18 | 3.393E+20 | 4.554E+20 | 2.878E+21 | 5.82E+19 | 1724.356 |
| 1987 | 9.07594E+21 | 3.14484E+20 | 7.14717E+21 | 3.57054E+21 | 3.385E+21 | 1.686E+22 | 3.634E+20 | 4.059E+20 | 1.2E+21 | 4.602E+19 | 1.485E+21 | 1.32E+19 | 1.771E+21 | 6.973E+19 | 3.578E+20 | 1.091E+19 | 3.403E+20 | 6.201E+20 | 3.158E+21 | 6.467E+19 | 1760.138 |
| 1988 | 8.96412E+21 | 3.1061E+20 | 7.78521E+21 | 3.5436E+21 | 3.609E+21 | 1.635E+22 | 3.45E+20 | 3.775E+20 | 1.2E+21 | 4.295E+19 | 1.631E+21 | 9.9E+18 | 1.183E+21 | 6.03E+19 | 2.727E+20 | 8.729E+18 | 3.427E+20 | 4.51E+20 | 3.418E+21 | 7.154E+19 | 1856.651 |
| 1989 | 8.99418E+21 | 3.11652E+20 | 7.93212E+21 | 3.55313E+21 | 3.14E+21 | 1.639E+22 | 4.25E+20 | 3.972E+20 | 1.2E+21 | 4.295E+19 | 1.441E+21 | 9.9E+18 | 1.134E+21 | 2.789E+19 | 2.306E+20 | 8.729E+18 | 3.496E+20 | 8.635E+20 | 3.694E+21 | 6.573E+19 | 1862.007 |
| 1990 | 9.12588E+21 | 3.16215E+20 | 9.64606E+21 | 3.55325E+21 | 3.635E+21 | 1.667E+22 | 3.049E+20 | 3.905E+20 | 1.253E+21 | 4.234E+19 | 1.838E+21 | 1.023E+19 | 1.738E+21 | 2.994E+19 | 2.065E+20 | 6.983E+18 | 3.315E+20 | 1.581E+21 | 3.817E+21 | 6.529E+19 | 2106.695 |
| 1991 | 9.12772E+21 | 3.16279E+20 | 1.00525E+22 | 3.55238E+21 | 3.197E+21 | 1.808E+22 | 3.542E+20 | 4.046E+20 | 1.14E+21 | 4.295E+19 | 1.725E+21 | 1.122E+19 | 2.039E+21 | 2.912E+19 | 2.466E+20 | 9.384E+18 | 3.331E+20 | 1.176E+21 | 3.864E+21 | 6.367E+19 | 2036.552 |
| 1992 | 8.90965E+21 | 3.08722E+20 | 1.00521E+22 | 3.51661E+21 | 3.372E+21 | 1.64E+22 | 4.25E+20 | 3.929E+20 | 1.278E+21 | 4.878E+19 | 1.896E+21 | 1.188E+19 | 1.613E+21 | 2.379E+19 | 2.606E+20 | 1.244E+19 | 3.102E+20 | 8.95E+20 | 4.554E+21 | 6.663E+19 | 2017.952 |
| 1993 | 8.25456E+21 | 2.86023E+20 | 9.43128E+21 | 3.47581E+21 | 2.592E+21 | 1.535E+22 | 3.912E+20 | 3.72E+20 | 1.572E+21 | 5.829E+19 | 1.43E+21 | 1.32E+19 | 1.567E+21 | 1.723E+19 | 2.307E+20 | 6.547E+18 | 3.472E+20 | 2.391E+21 | 4.267E+21 | 6.824E+19 | 1838.951 |
| 1994 | 8.03714E+21 | 2.7849E+20 | 9.89371E+21 | 3.42308E+21 | 2.02E+21 | 1.528E+22 | 3.881E+20 | 3.517E+20 | 1.758E+21 | 7.21E+19 | 1.256E+21 | 1.122E+19 | 1.5E+21 | 1.026E+19 | 2.164E+20 | 2.837E+18 | 3.688E+20 | 2.582E+21 | 4.329E+21 | 5.965E+19 | 1881.087 |
| 1995 | 8.26587E+21 | 2.86415E+20 | 1.12991E+22 | 3.39422E+21 | 2.082E+21 | 1.538E+22 | 4.374E+20 | 4.36E+20 | 1.794E+21 | 7.21E+19 | 1.863E+21 | 1.122E+19 | 1.692E+21 | 5.743E+18 | 2.102E+20 | 3.055E+18 | 3.706E+20 | 2.931E+21 | 3.957E+21 | 5.697E+19 | 2057.697 |
| 1996 | 8.32219E+21 | 2.88367E+20 | 1.16651E+22 | 3.37142E+21 | 2.487E+21 | 1.611E+22 | 4.22E+20 | 4.835E+20 | 1.938E+21 | 7.056E+19 | 1.946E+21 | 1.32E+19 | 1.852E+21 | 1.108E+19 | 1.974E+20 | 4.365E+18 | 3.997E+20 | 2.991E+21 | 2.708E+21 | 5.524E+19 | 2092.494 |
| 1997 | 8.26189E+21 | 2.86277E+20 | 1.18969E+22 | 3.3457E+21 | 2.768E+21 | 1.562E+22 | 4.343E+20 | 4.902E+20 | 2.076E+21 | 7.67E+19 | 1.812E+21 | 1.32E+19 | 1.289E+21 | 9.845E+18 | 2.037E+20 | 6.547E+18 | 4.028E+20 | 3.399E+21 | 3.068E+21 | 5.675E+19 | 2073.055 |
| 1998 | 8.20634E+21 | 2.84353E+20 | 1.1807E+22 | 3.2959E+21 | 2.036E+21 | 1.524E+22 | 4.589E+20 | 5.044E+20 | 2.142E+21 | 9.204E+19 | 1.265E+21 | 1.386E+19 | 1.757E+21 | 3.282E+18 | 2.337E+20 | 5.892E+18 | 4.543E+20 | 3.146E+21 | 3.372E+21 | 6.317E+19 | 1984.629 |
| 1999 | 8.17137E+21 | 2.83141E+20 | 1.21288E+22 | 3.3273E+21 | 2.454E+21 | 1.429E+22 | 5.174E+20 | 5.648E+20 | 2.334E+21 | 1.037E+20 | 1.978E+21 | 1.617E+19 | 1.094E+21 | 1.231E+18 | 3.485E+20 | 5.456E+18 | 5.074E+20 | 3.746E+21 | 3.027E+21 | 6.568E+19 | 1985.808 |
| 2000 | 7.53472E+21 | 2.61081E+20 | 1.20772E+22 | 4.16726E+21 | 1.786E+21 | 1.249E+22 | 6.252E+20 | 4.829E+20 | 2.724E+21 | 1.104E+20 | 1.721E+21 | 1.65E+19 | 7.905E+20 | 8.204E+17 | 4.389E+20 | 6.547E+18 | 6.615E+20 | 3.327E+21 | 3.077E+21 | 6.495E+19 | 1859.139 |
| 2001 | 6.98224E+21 | 2.41937E+20 | 1.26529E+22 | 4.00894E+21 | 1.167E+21 | 1.105E+22 | 6.499E+20 | 4.631E+20 | 2.724E+21 | 1.289E+20 | 2.107E+21 | 1.65E+19 | 8.555E+20 | 8.204E+17 | 4.076E+20 | 8.729E+18 | 7.354E+20 | 4.515E+21 | 3.569E+21 | 6.729E+19 | 1801.766 |
| 2002 | 6.68209E+21 | 2.31537E+20 | 1.26491E+22 | 3.31024E+21 | 8.358E+20 | 9.835E+21 | 6.868E+20 | 3.578E+20 | 2.514E+21 | 1.381E+20 | 1.65E+21 | 1.98E+19 | 6.064E+20 | 8.204E+17 | 3.496E+20 | 1.091E+19 | 7.944E+20 | 4.378E+21 | 3.197E+21 | 7.728E+19 | 1739.557 |
| 2003 | 6.27432E+21 | 2.17408E+20 | 1.23027E+22 | 3.70759E+21 | 6.548E+20 | 8.161E+21 | 6.622E+20 | 3.147E+20 | 2.502E+21 | 1.411E+20 | 1.52E+21 | 1.65E+19 | 5.685E+20 | 8.204E+17 | 2.959E+20 | 1.091E+19 | 8.011E+20 | 4.968E+21 | 2.56E+21 | 7.405E+19 | 1621.194 |
| 2004 | 6.19262E+21 | 2.14576E+20 | 1.25943E+22 | 3.7329E+21 | 6.008E+20 | 8.665E+21 | 6.93E+20 | 3.363E+20 | 2.461E+21 | 1.442E+20 | 1.713E+21 | 1.98E+19 | 6.172E+20 | 4.102E+17 | 2.9E+20 | 1.091E+19 | 7.874E+20 | 5.525E+21 | 2.722E+21 | 7.739E+19 | 1676.360 |
| 2005 | 6.33245E+21 | 2.19422E+20 | 1.2703E+22 | 3.51347E+21 | 6.934E+20 | 8.134E+21 | 7.977E+20 | 3.819E+20 | 2.844E+21 | 1.503E+20 | 1.76E+21 | 1.98E+19 | 5.848E+20 | 4.102E+17 | 2.861E+20 | 8.729E+18 | 7.838E+20 | 5.052E+21 | 2.762E+21 | 8.058E+19 | 1689.288 |
| 2006 | 6.05674E+21 | 2.09868E+20 | 1.31001E+22 | 3.32758E+21 | 5.671E+20 | 8.608E+21 | 2.895E+20 | 1.343E+20 | 1.656E+21 | 1.012E+20 | 1.184E+21 | 1.98E+19 | 6.443E+20 | 4.102E+17 | 2.81E+20 | 8.729E+18 | 7.725E+20 | 5.739E+21 | 3.12E+21 | 8.504E+19 | 1692.757 |
| 2007 | 6.13925E+21 | 2.12727E+20 | 1.29726E+22 | 3.22347E+21 | 6.276E+20 | 8.034E+21 | 3.08E+20 | 1.349E+20 | 1.692E+21 | 1.104E+20 | 1.071E+21 | 1.98E+19 | 6.876E+20 | 4.102E+17 | 2.676E+20 | 6.547E+18 | 7.731E+20 | 6.131E+21 | 3.115E+21 | 8.939E+19 | 1651.026 |
| 2008 | 5.85464E+21 | 2.02865E+20 | 1.2606E+22 | 3.11875E+21 | 6.923E+20 | 8.331E+21 | 3.419E+20 | 2.402E+20 | 1.836E+21 | 1.595E+20 | 1.392E+21 | 2.31E+19 | 7.634E+20 | 4.102E+17 | 2.443E+20 | 6.547E+18 | 7.901E+20 | 6.537E+21 | 2.658E+21 | 9.056E+19 | 1619.058 |
| 2009 | 5.88229E+21 | 2.03824E+20 | 1.27291E+22 | 3.0627E+21 | 7.645E+20 | 8.41E+21 | 3.588E+20 | 2.577E+20 | 1.872E+21 | 1.654E+20 | 1.458E+21 | 2.739E+19 | 7.607E+20 | 2.461E+17 | 2.339E+20 | 8.075E+18 | 7.941E+20 | 6.227E+21 | 2.21E+21 | 9.341E+19 | 1623.576 |
| 2010 | 5.73599E+21 | 1.98754E+20 | 1.29725E+22 | 2.96477E+21 | 7.806E+20 | 8.177E+21 | 3.758E+20 | 2.544E+20 | 1.825E+21 | 1.657E+20 | 1.312E+21 | 2.776E+19 | 7.959E+20 | 1.657E+17 | 2.307E+20 | 6.983E+18 | 8.17E+20 | 6.226E+21 | 2.071E+21 | 9.095E+19 | 1646.843 |
| 2011 | 6.03574E+21 | 2.09141E+20 | 1.27592E+22 | 2.85155E+21 | 8.333E+20 | 8.188E+21 | 4.494E+20 | 2.796E+20 | 1.897E+21 | 1.644E+20 | 1.323E+21 | 2.99E+19 | 8.76E+20 | 1.251E+17 | 2.101E+20 | 6.423E+18 | 8.17E+20 | 6.226E+21 | 2.113E+21 | 9.471E+19 | 1620.101 |
| 2012 | 5.6745E+21 | 1.96624E+20 | 1.27798E+22 | 2.79433E+21 | 8.194E+20 | 7.674E+21 | 8.963E+20 | 3.4E+20 | 2.196E+21 | 1.626E+20 | 1.264E+21 | 2.884E+19 | 8.122E+20 | 1.231E+17 | 2.062E+20 | 5.674E+18 | 8.189E+20 | 6.151E+21 | 1.974E+21 | 9.765E+19 | 1611.427 |
| 2013 | 5.65514E+21 | 1.95953E+20 | 1.31257E+22 | 2.78698E+21 | 8.276E+20 | 7.319E+21 | 8.242E+20 | 3.219E+20 | 2.028E+21 | 1.592E+20 | 1.247E+21 | 3.037E+19 | 7.569E+20 | 1.067E+17 | 2.101E+20 | 5.456E+18 | 7.939E+20 | 6.255E+21 | 1.786E+21 | 9.408E+19 | 1621.774 |
| 2014 | 5.63179E+21 | 1.95144E+20 | 1.30238E+22 | 2.77915E+21 | 8.925E+20 | 7.444E+21 | 9.271E+20 | 3.534E+20 | 2.148E+21 | 1.227E+20 | 1.02E+21 | 2.495E+19 | 6.768E+20 | 9.763E+16 | 2.062E+20 | 3.71E+18 | 7.933E+20 | 6.247E+21 | 1.521E+21 | 9.207E+19 | 1599.020 |

**Table C in S1 File** Emergy and carbon emissions-based production efficiency indicators

| **Year** | **U (sej)** | **Sow area**  **(10^3^ ha)** | **EID**  **(sej/ha)** | **ELR** | **PIR** | **SSR** | **EYR** | **Output value**  **(10^8^ RMB)** |
| --- | --- | --- | --- | --- | --- | --- | --- | --- |
| 1978 | 1.74824E+22 | 4760.13 | 3.67269E+15 | 0.333 | 0.779 | 0.562 | 3.316 | 48.86 |
| 1979 | 1.76551E+22 | 4731.60 | 3.73132E+15 | 0.347 | 0.803 | 0.555 | 3.469 | 53.77 |
| 1980 | 1.83657E+22 | 4685.71 | 3.91951E+15 | 0.409 | 0.888 | 0.530 | 3.026 | 50.53 |
| 1981 | 1.83785E+22 | 4644.13 | 3.95735E+15 | 0.418 | 0.901 | 0.526 | 3.003 | 50.99 |
| 1982 | 1.88167E+22 | 4626.00 | 4.0676E+15 | 0.449 | 0.947 | 0.514 | 3.365 | 60.41 |
| 1983 | 1.90632E+22 | 4578.07 | 4.164E+15 | 0.476 | 0.988 | 0.503 | 3.004 | 56.10 |
| 1984 | 1.90779E+22 | 4526.87 | 4.21425E+15 | 0.486 | 1.006 | 0.499 | 3.478 | 64.69 |
| 1985 | 1.92601E+22 | 4451.70 | 4.32616E+15 | 0.511 | 1.046 | 0.489 | 3.153 | 61.52 |
| 1986 | 1.96575E+22 | 4361.80 | 4.50653E+15 | 0.565 | 1.114 | 0.473 | 2.930 | 62.00 |
| 1987 | 2.01081E+22 | 4374.27 | 4.59688E+15 | 0.590 | 1.141 | 0.467 | 2.814 | 61.39 |
| 1988 | 2.06035E+22 | 4300.50 | 4.79096E+15 | 0.647 | 1.221 | 0.450 | 2.592 | 59.92 |
| 1989 | 2.07911E+22 | 4312.87 | 4.82034E+15 | 0.657 | 1.234 | 0.448 | 2.562 | 60.85 |
| 1990 | 2.26414E+22 | 4384.69 | 5.15739E+15 | 0.786 | 1.398 | 0.417 | 2.418 | 62.95 |
| 1991 | 2.30488E+22 | 4379.53 | 5.26229E+15 | 0.818 | 1.441 | 0.410 | 2.404 | 67.26 |
| 1992 | 2.27871E+22 | 4275.05 | 5.33007E+15 | 0.834 | 1.472 | 0.405 | 2.326 | 63.03 |
| 1993 | 2.14477E+22 | 3926.16 | 5.46271E+15 | 0.828 | 1.511 | 0.398 | 2.376 | 62.29 |
| 1994 | 2.16324E+22 | 3802.42 | 5.68975E+15 | 0.888 | 1.601 | 0.384 | 2.268 | 62.28 |
| 1995 | 2.32456E+22 | 3923.04 | 5.92541E+15 | 0.994 | 1.718 | 0.368 | 2.130 | 66.03 |
| 1996 | 2.36471E+22 | 3963.82 | 5.96546E+15 | 1.022 | 1.746 | 0.364 | 2.108 | 69.65 |
| 1997 | 2.37908E+22 | 3944.16 | 6.03185E+15 | 1.050 | 1.783 | 0.359 | 2.081 | 69.63 |
| 1998 | 2.35936E+22 | 3919.60 | 6.01877E+15 | 1.051 | 1.779 | 0.360 | 2.038 | 69.94 |
| 1999 | 2.39106E+22 | 3899.49 | 6.1317E+15 | 1.079 | 1.828 | 0.354 | 2.009 | 75.03 |
| 2000 | 2.40402E+22 | 3554.33 | 6.7637E+15 | 1.054 | 2.084 | 0.324 | 1.744 | 73.33 |
| 2001 | 2.3886E+22 | 3245.93 | 7.35875E+15 | 1.173 | 2.306 | 0.302 | 1.708 | 78.54 |
| 2002 | 2.2873E+22 | 3064.54 | 7.46385E+15 | 1.289 | 2.308 | 0.302 | 1.595 | 83.73 |
| 2003 | 2.2502E+22 | 2834.39 | 7.9389E+15 | 1.254 | 2.466 | 0.288 | 1.452 | 88.33 |
| 2004 | 2.27344E+22 | 2778.41 | 8.18256E+15 | 1.291 | 2.548 | 0.282 | 1.511 | 92.83 |
| 2005 | 2.27684E+22 | 2837.94 | 8.02296E+15 | 1.312 | 2.475 | 0.288 | 1.501 | 95.34 |
| 2006 | 2.26943E+22 | 2686.26 | 9.03651E+15 | 1.418 | 2.621 | 0.276 | 1.413 | 98.86 |
| 2007 | 2.2548E+22 | 2643.23 | 9.15544E+15 | 1.408 | 2.550 | 0.282 | 1.424 | 101.43 |
| 2008 | 2.17823E+22 | 2482.43 | 8.77469E+15 | 1.427 | 2.596 | 0.278 | 1.533 | 106.38 |
| 2009 | 2.18779E+22 | 2504.79 | 8.73438E+15 | 1.446 | 2.595 | 0.278 | 1.497 | 109.57 |
| 2010 | 2.1872E+22 | 2484.65 | 8.80267E+15 | 1.514 | 2.685 | 0.271 | 1.453 | 110.55 |
| 2011 | 2.18556E+22 | 2642.70 | 8.87462E+15 | 1.459 | 2.500 | 0.286 | 1.506 | 112.60 |
| 2012 | 2.14453E+22 | 2450.48 | 9.22696E+15 | 1.532 | 2.653 | 0.274 | 1.505 | 113.87 |
| 2013 | 2.17638E+22 | 2443.43 | 9.41365E+15 | 1.578 | 2.720 | 0.269 | 1.424 | 114.73 |
| 2014 | 2.16299E+22 | 2414.02 | 9.51183E+15 | 1.572 | 2.712 | 0.269 | 1.422 | 118.86 |

Note: All monetary output value is converted to 1978 prices based on the price index.

| **Table D in S1 File**  The inputs and outputs data in DEA | | | | | | | |  |
| --- | --- | --- | --- | --- | --- | --- | --- | --- |
| Year | Prefecture | Labor input (10^4^ persons) | Sown land (million ha.) | Nitrogen Fertilizer  (10^4^ tons) | Mechanical Power  (million kW) | Value added  (100 million RMB) | CO_2-eq_  (10^4^ tons) |  |
| 1995 | Hangzhou | 117.48 | 499.6 | 9.01 | 221.56 | 467800 | 261.99 |  |
|  | Ningbo | 100.18 | 512.1 | 8.39 | 206.5 | 511133 | 268.53 |  |
|  | Wenzhou | 60.63 | 366.24 | 6.8 | 155.93 | 55200 | 192.05 |  |
|  | jiaxing | 49.07 | 453.9 | 10.35 | 179.9 | 120200 | 238.02 |  |
|  | Huzhou | 92.69 | 295.54 | 6.35 | 150.46 | 276200 | 154.98 |  |
|  | Shaoxing | 12.85 | 447.89 | 6.53 | 148.88 | 17800 | 234.87 |  |
|  | Jinhua | 128.07 | 420.8 | 6.86 | 125.1 | 382000 | 220.67 |  |
|  | Quzhou | 128.89 | 265.6 | 4.46 | 51.1 | 216700 | 139.28 |  |
|  | Zhoushan | 63.03 | 43.7 | 0.45 | 138.74 | 105200 | 22.912 |  |
|  | Taizhou | 129.68 | 401 | 6.29 | 200.06 | 188600 | 210.24 |  |
|  | Lishui | 67.25 | 217.5 | 2.81 | 61.9 | 200900 | 114.05 |  |
| 2000 |  |  |  |  |  |  |  |  |
|  | Hangzhou | 96.94 | 449.69 | 7.2345 | 266.42 | 649600 | 235.21 |  |
|  | Ningbo | 84.04 | 445.9 | 7.41 | 190.2 | 539724 | 233.23 |  |
|  | Wenzhou | 47.66 | 337.17 | 5.691192 | 189.36 | 278000 | 176.36 |  |
|  | jiaxing | 42.52 | 408.5 | 8.64 | 197.5 | 396500 | 213.67 |  |
|  | Huzhou | 79.11 | 248.35 | 3.0177 | 168.41 | 305300 | 129.90 |  |
|  | Shaoxing | 11.3 | 408.79 | 8.1039 | 189.86 | 487800 | 213.82 |  |
|  | Jinhua | 114.37 | 379.9 | 6.7142 | 160.1 | 367700 | 198.71 |  |
|  | Quzhou | 123.12 | 251.8 | 3.886 | 57.5 | 216100 | 131.70 |  |
|  | Zhoushan | 56.59 | 35.2 | 0.2772 | 168.1 | 44400 | 18.41 |  |
|  | Taizhou | 112.07 | 377.45 | 6.3201 | 255.76 | 415600 | 197.39 |  |
|  | Lishui | 64.14 | 211.6 | 2.365208 | 68 | 200900 | 110.68 |  |
| 2005 |  |  |  |  |  |  |  |  |
|  | Hangzhou | 70.64 | 397.6 | 7.1775 | 297.3 | 840900 | 233.04 |  |
|  | Ningbo | 61.6 | 332.53 | 6.6 | 284.4 | 706600 | 194.90 |  |
|  | Wenzhou | 30.14 | 276.02 | 5.32704 | 198.1 | 321000 | 161.78 |  |
|  | jiaxing | 27 | 354.2 | 8.176 | 172 | 478800 | 207.61 |  |
|  | Huzhou | 56.32 | 233.11 | 3.528 | 153.54 | 291200 | 136.63 |  |
|  | Shaoxing | 6.76 | 300.9 | 7.0302 | 202.22 | 596700 | 176.36 |  |
|  | Jinhua | 99.21 | 262.2 | 5.7126 | 192.9 | 407800 | 153.68 |  |
|  | Quzhou | 100.05 | 216.9 | 4.928 | 82.5 | 306800 | 127.13 |  |
|  | Zhoushan | 49.1 | 27.23 | 0.2646 | 163.6 | 39200 | 15.95 |  |
|  | Taizhou | 87.17 | 286.01 | 5.3054 | 288.63 | 466400 | 167.59 |  |
|  | Lishui | 53.32 | 195.43 | 2.06066 | 72.3042 | 329800 | 114.54 |  |
| 2010 |  |  |  |  |  |  |  |  |
|  | Hangzhou | 55.68 | 381.83 | 5.85 | 322 | 1219343 | 223.36 |  |
|  | Ningbo | 47.41 | 318.6 | 5.51 | 327.87 | 1243700 | 186.35 |  |
|  | Wenzhou | 24.45 | 256.3 | 5.23 | 229.82 | 468800 | 149.92 |  |
|  | jiaxing | 18.12 | 340.2 | 7.84 | 158.6 | 715100 | 199.01 |  |
|  | Huzhou | 43.18 | 224.8 | 3.34 | 163.1 | 537700 | 131.48 |  |
|  | Shaoxing | 4.72 | 329.8 | 7.2 | 246.12 | 998300 | 192.93 |  |
|  | Jinhua | 79.98 | 273.7 | 5.39 | 246.5 | 681500 | 160.11 |  |
|  | Quzhou | 79.29 | 223.62 | 3.96 | 158.9 | 357500 | 130.81 |  |
|  | Zhoushan | 43.77 | 24.11 | 0.26 | 171.91 | 60700 | 14.10 |  |
|  | Taizhou | 58.68 | 265.43 | 5.01 | 360 | 737700 | 155.23 |  |
|  | Lishui | 46.39 | 176.9 | 2.91 | 103.3 | 458300 | 103.48 |  |
| 2014 |  |  |  |  |  |  |  |  |
|  | Hangzhou | 49.74 | 294.96 | 4.68 | 342.19 | 1685700 | 197.22 |  |
|  | Ningbo | 42.71 | 286.61 | 4.65 | 312.77 | 1557045 | 191.64 |  |
|  | Wenzhou | 22.84 | 218.76 | 4.78 | 219.17 | 643000 | 146.27 |  |
|  | jiaxing | 14.67 | 312.81 | 7.81 | 147.87 | 891200 | 209.16 |  |
|  | Huzhou | 42.48 | 187.12 | 2.92 | 168.47 | 627400 | 125.12 |  |
|  | Shaoxing | 4.63 | 268.81 | 7.25 | 230.92 | 1323100 | 179.74 |  |
|  | Jinhua | 76.35 | 226.32 | 4.44 | 261.41 | 925900 | 151.33 |  |
|  | Quzhou | 74.46 | 207.12 | 3.84 | 162.95 | 472500 | 138.49 |  |
|  | Zhoushan | 41.76 | 13.45 | 0.24 | 159.37 | 70900 | 14.06 |  |
|  | Taizhou | 57.63 | 203.6 | 4.41 | 316.72 | 932800 | 136.09 |  |
|  | Lishui | 43.2 | 164.29 | 2.61 | 112.49 | 649300 | 109.85 |  |

Note: All the input and output data of crop production were drawn from local statistical year books in the 11 prefectures and Zhejiang Province [44-54]. Some missing data were estimated through cross-reference and proportional calculation.

Reference:

44. HANGZHOU STATISTICAL YEARBOOK(1995-2016), Hangzhou bureau of statistics China Statistics Press, Beijing.

45. LISHUI STATISTICAL YEARBOOK (2001-2014), LISHUI bureau of statistics, China Statistics Press, Beijing.

46. QUZHOU STATISTICAL YEARBOOK (2007-2016), QUZHOU bureau of statistics, China Statistics Press, Beijing.

47. TAIZHOU STATISTICAL YEARBOOK (1998-2016), TAIZHOU bureau of statistics, China Statistics Press, Beijing.

48. ZHOUSHAN STATISTICAL YEARBOOK (1997-2016) , ZHOUSHAN bureau of statistics, China Statistics Press, Beijing.

49. JINHUA STATISTICAL YEARBOOK (1996-2016), JINHUA bureau of statistics, China Statistics Press, Beijing.

50. JIAXING STATISTICAL YEARBOOK (1997-2016), JIAXING bureau of statistics, China Statistics Press, Beijing.

51. NINGBO Statistical YearBook (1996-2016), NINGBO bureau of statistics, China Statistics Press, Beijing.

52. SHAOXING STATISTICAL YEARBOOK (1995-2016), SHAOXING bureau of statistics, China Statistics Press, Beijing.

53. Wenzhou Statistical Yearbook (1998-2016), Wenzhou bureau of statistics, China Statistics Press

54. HUZHOU STATISTICAL YEARBOOK (2002-2016), HUZHOU bureau of statistics, China Statistics Press, Beijing.
